# Influencing Factors and Regulatory Mechanisms of Fresh Tea Leaf Quality: A Review

**DOI:** 10.3390/foods14183268

**Published:** 2025-09-20

**Authors:** Tianyu Wu, Junjie He, Xiujuan Deng, Xiaohua Wang, Wenxia Yuan, Qiaomei Wang, Xinya Chen, Man Zou, Hongmei An, Baijuan Wang, Raoqiong Che

**Affiliations:** 1 College of Tea Science, Yunnan Agricultural University, Kunming 650201, China; wty15508851042@163.com (T.W.); 15808869561@163.com (X.D.); m17628681243@163.com (X.W.); 2008002@ynau.edu.cn (W.Y.); wqm19850127@163.com (Q.W.); chenxinya128@163.com (X.C.); 15770383235@163.com (M.Z.); 2 Yunnan Organic Tea Industry Intelligent Engineering Research Center, Kunming 650201, China; 18087827443@163.com (J.H.); 18087350784@163.com (H.A.)

**Keywords:** tea plant, quality, variety, environmental factor, secondary metabolites

## Abstract

The contents of secondary metabolites such as tea polyphenols, amino acids, caffeine, and volatile metabolites in fresh tea leaves are key factors determining the unique flavor and health attributes of finished tea products. However, differences in varieties, cultivation practices, and environmental conditions often lead to variations in these metabolites among fresh tea leaves, thereby affecting tea quality. In order to clarify the various internal and external factors that influence the formation of the quality of fresh tea leaves and their mechanism of action. This article mainly reviews the research on fresh leaf quality in the past decade. Firstly, it clarifies the molecular basis of metabolic differences among varieties. Then, it summarizes the regulatory mechanisms of underground (soil, microorganisms) and above-ground (light, temperature, humidity) environments on key metabolic pathways, and focuses on evaluating the effects of intercropping, fertilization, and other cultivation measures on improving tea quality. This review found that the specific gene expression of varieties, the transmission of environmental signals, and cultivation interventions jointly drive the synthesis and accumulation of tea polyphenols, amino acids, caffeine, and aroma substances. However, no one has ever systematically reviewed it. Therefore, it provides certain theoretical references for improving the quality of fresh leaves.

## 1. Introduction

Tea is one of the oldest and most popular beverages in the world, widely loved for its unique flavor and health-promoting properties. It is expected that the global output will reach 7.4 million tons by 2025 [1]. Fresh tea leaves, as the raw material for finished tea products, serve as the material basis for the formation of tea quality, determining its final sensory and chemical characteristics. It has been demonstrated that variations in secondary metabolites—such as tea polyphenols, amino acids, and caffeine—in fresh leaves significantly influence the taste and overall quality of processed tea [2,3,4]. Previous studies have revealed that the astringency and bitterness of fresh leaves from albino tea cultivars are significantly lower than those from green-leaf tea cultivars, which is attributed to the marked differences in amino acid and flavonoid contents between the two types [4]. Research has revealed that the fresh leaves of the ‘Yinghong 9’ cultivar are characterized by relatively high levels of 3-hexen-1-ol, acetate, decanal, and geraniol, which contribute to the floral and fruity aroma of the finished tea product [5].

Like other plants, tea accumulates distinct secondary metabolites governed by cultivar, environment, and management [6]. Previous studies confirm these factors steer both the profile and abundance of fresh-leaf metabolites [7]. Previous studies have demonstrated that the types and contents of secondary metabolites in fresh tea leaves are predominantly influenced by key factors such as tea cultivar/variety, the growing environment, and cultivation management practices [7]. In terms of genetic factors, the synthesis capacities of amino acids, caffeine, tea polyphenols, and volatile components are significantly influenced by genotypic differences among tea plant varieties [8]. This genetic diversity is directly reflected in the sensory quality of the finished tea. For example, in the production of Liuan Guapian, ‘Anhui No. 1’ is characterized by a pronounced floral aroma and fresh taste, the population variety is distinguished by its unique roasted fragrance and mellow texture, whereas ‘Shuchazao’ presents a cooked corn-like aroma and bitter taste [9]. The influence of environmental factors on tea quality should also not be overlooked. Elevated altitude has been shown to promote the accumulation of amino acids and aromatic compounds (e.g., aldehydes, ketones, and methyl salicylate) while reducing the catechin content [10]. Soil microbial communities have been found to regulate L-theanine synthesis through nitrogen metabolism processes, whereas temperature variations have been demonstrated to significantly modulate the biosynthetic pathways of flavonoid compounds [11,12]. In addition to the aforementioned factors, cultivation management practices are crucial for regulating the quality of fresh tea leaves. Rational fertilization strategies can significantly enhance the contents of catechins, caffeine, and total amino acids in fresh leaves, thereby improving the overall quality of tea products [13].

At present, the metabolic pathways of key characteristic compounds in tea have been well characterized. However, the regulatory mechanisms through which the aforementioned factors influencing fresh tea leaf quality modulate these metabolic pathways remain poorly understood. Furthermore, exploring how to regulate these influencing factors to improve tea quality and thereby reduce the use of chemical fertilizers and pesticides is an important research direction for green agricultural development. In order to clarify how these factors regulate specialized-metabolite synthesis in fresh tea leaves, we reviewed the past decade of research. It summarized new findings on leaf-quality components. Whole-genome, transcriptomic, proteomic, and metabolomic tools were covered (Table S2). These technologies identify the influencing factors and mechanisms of fresh tea leaf quality. This study aims to provide valuable insights into the precise modulation of secondary metabolite synthesis in fresh tea leaves through optimized tea plant varieties (or cultivars), rational selection of growth environments, and scientifically implemented cultivation management techniques, ultimately enhancing the quality of fresh tea leaves.

## 2. The Impact of Genetic Factors on Fresh Tea Leaf Quality and the Underlying Metabolic Mechanisms

Owing to their distinct genetic backgrounds, different tea plant varieties exhibit significant variations in the synthesis and accumulation of secondary metabolites such as amino acids, caffeine, tea polyphenols, and volatile compounds, as well as in leaf color and aroma characteristics. These differences consequently lead to diverse quality attributes in fresh tea leaves [14,15]. To meet the diverse market demands for the health benefits and flavor quality of tea, tea germplasm resources with unique chemical composition characteristics are being systematically developed and utilized (Table 1).

### 2.1. The Impact of Genetic Factors on Polyphenols in Fresh Tea Leaves and the Underlying Metabolic Mechanisms

**Figure 1:**
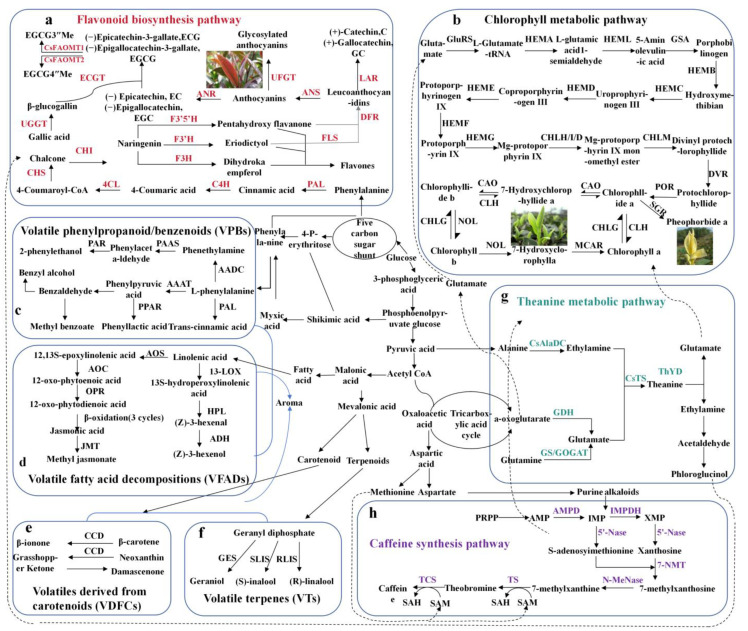
Biosynthesis and metabolic pathways of secondary metabolites in tea plants: (**a**) flavonoid biosynthesis pathway; (**b**) chlorophyll metabolic pathway; (**c**–**f**) synthetic metabolic pathways of volatile compounds in tea; (**g**) theanine metabolic pathway; (**h**) caffeine synthesis pathway.

These differentially abundant metabolites primarily include tea polyphenols, caffeine, amino acids, etc. The polyphenolic compounds in tea plants are primarily classified into four major categories: catechins, flavonoids and flavonoid glycosides, phenolic acids and depsides, as well as anthocyanins and leucoanthocyanidins (Figure 1). Among these, catechins account for approximately 70% of the total tea polyphenols. Nonesterified catechins are synthesized from phenylalanine through a series of enzymatic reactions, whereas esterified catechins are generated via the galloylation of nonesterified catechins. The study revealed that the key enzymes involved in the synthesis of nonesterified catechins, including dihydroflavonol 4-reductase (DFR), leucoanthocyanidin reductase (LAR), anthocyanidin synthase (ANS), and anthocyanidin reductase (ANR), are located at the terminal end of the flavonoid metabolic pathway [24,25] (Figure 1a). These differences in enzyme activities and gene expression among varieties collectively determine the composition and content of catechins. Polymorphism of the DFR gene leads to differences in the composition and content of nonesterified catechins between Camellia sinensis var. sinensis and Camellia sinensis var. Assamica [24]. In tobacco, the overexpression of the DFR gene was found to increase the content of catechins such as epicatechin (EC) and epigallocatechin (EGC) [25]. The high activity and expression levels of LAR and ANS are also considered to be the reasons for the elevated gallocatechin (GC) and catechins (C) contents in certain tea plant varieties [14]. Research has demonstrated that the EC content is positively correlated with the expression levels of LAR and ANS genes [26]. Additionally, the activity of the ANR gene and its regulatory mechanisms in tea plants play crucial roles in the biosynthesis of nongallated catechins. Zhang et al. [27] reported that different ANR genotypes among tea plant subpopulations lead to variations in catechin composition. Higher levels of EC, EGC, and C in ‘Shuchazao’ were confirmed to be associated with elevated ANR expression [28]. Furthermore, it was revealed that the R2R3-MYB transcription factor TT2 promotes the accumulation of GC, EGC, and EC in cultivars such as ‘Lingtou Dancong’ by activating ANR gene expression [29]. In addition, the synthesis of nonesterified catechins can also be influenced by enzymes such as phenylalanine ammonia lyase (PAL), cinnamate 4-hydroxylase (C4H), 4-coumaroyl-CoA ligase (4CL), chalcone synthase (CHS), chalcone isomerase (CHI), flavanone 3-hydroxylase (F3H), flavonoid 3′-hydroxylase (F3’H), and flavonoid 3′,5′-hydroxylase (F3’5’H) [27]. Higher expression levels of the PAL and C4H genes were observed in the cultivars ‘UPASI 10’, ‘AV-2’, and ‘HV-39’, which contain higher catechin contents [30].

The conversion of nongalloylated catechins to galloylated catechins in tea plants is mediated primarily through galloylation, which involves two key enzymes: galloyl-1-O-β-d-glucosyltransferase (UGGT) and 1-O-galloyl-β-d-glucose O-galloyltransferase (ECGT) (Figure 1a). In the tea cultivars ‘Baitangshan Tea’ and ‘Baitang Ziya Tea,’ the UGGT genes (CSS0004941, CSS0009705, CSS0023751, and CSS0031621) were found to be significantly upregulated, accompanied by a notable increase in the content of galloylated catechins [8]. Interestingly, different UGGT isoforms in tea plants catalyze distinct precursor substrates; for instance, UGT75L12 has been demonstrated to glycosylate various flavonoids [31]. The serine carboxypeptidase-like (SCPL) family contributes to the synthesis of galloylated catechins either through enzymatic catalysis or noncatalytic chaperone functions. Among the SCPL family members, SCPL4 exhibits catalytic activity, whereas SCPL5 functions as a chaperone, with expression levels positively correlated with galloylated catechin accumulation [27,32]. Additionally, the expression pattern of SCPL1A aligns with the accumulation of galloylated catechins [8]. Further studies identified UGT75E2/3 and SCPL1/3 as potential participants in catechin galloylation [33]. The types and contents of galloylated catechins in tea leaves are also influenced by regulatory factors that modulate genes related to catechin galloylation. For example, MYB1 was confirmed to upregulate the transcription of ANS, ANR, and SCPL, leading to significantly higher galloylated catechin levels in cultivated tea varieties than in wild-type plants [34]. The ratio of nongalloylated to galloylated catechins in tea leaves is not solely determined by biosynthetic enzymes but is also affected by hydrolytic enzymes. Tannase (TA), which hydrolyzes galloylated catechins into nongalloylated forms, is inversely correlated with the galloylated catechin content, thereby modulating the balance between the two catechin classes [35].

### 2.2. The Impact of Genetic Factors on Leaf Color in Fresh Tea Leaves and the Underlying Metabolic Mechanisms

The leaf color germplasm resources of tea primarily consist of two categories: purple-leaf tea and albino leaf tea. Notably, purple-leaf tea is characterized by a high anthocyanin content, which is regulated by specific genes within the tea tree [36] (Figure 1a). Anthocyanins, which are flavonoids, are synthesized from phenylalanine through catalysis by enzymes such as PAL, CHS, and F3H to generate colorless anthocyanin precursors (Figure 1a). Subsequently, unstable colored anthocyanins and their stable glycosylated forms (e.g., anthocyanin-3-glucoside) are formed via sequential catalysis by ANS and UFGT. The high expression of these enzyme genes is regarded as the primary factor contributing to anthocyanin accumulation and the resultant purple coloration in tea leaves. Extensive studies have demonstrated that in purple tea leaves, the upregulated expression of genes encoding enzymes such as PAL, CHS, F3H, ANS, 4CL, and UFGT promotes anthocyanin accumulation, leading to purple leaf coloration [37,38]. Additionally, it has been reported that transcription factors such as MYB, bHLH, and WD40 can form protein complexes that regulate anthocyanin accumulation in tea plants by binding to the promoter regions of anthocyanin biosynthetic genes [36,39]. Currently, the differences in secondary metabolites between purple and green tea plant varieties remain inconclusive. Most scholars believe that purple tea varieties present relatively high levels of anthocyanins, flavonoids, and total catechins. However, some studies have reported that the catechin content in ‘Zijuan’ is lower than that in green varieties [36,37,38,39,40,41]. This discrepancy may be attributed to variations in the experimental materials. For example, the purple coloration of ‘Zijuan’ leaves is associated primarily with an increase in flavonoids/anthocyanins, whereas in ‘Zixuan’, it is regulated by multiple pathways, including weakened chlorophyll metabolism and reduced carotenoid content [42].

The coloration of albino tea is typically white or yellow, which can be classified into three albinism types: ecologically sensitive, ecologically insensitive, and ecologically composite. Studies have shown that tea leaf albinism is directly related to a reduction in chlorophyll content; when the expression of genes encoding chlorophyll synthesis-related enzymes (such as HEMA, POR, CRD, and DVR) is downregulated, the leaf color may turn white [43,44]. Additionally, some studies have suggested that the lower chlorophyll content in albino tea could be attributed to extensive chlorophyll degradation. Researchers have reported that regulatory genes (SGR) and chlorophyll degradation-related genes (NOL and SGR) are highly expressed in albino tea [44]. Interestingly, during the whitening process of fresh tea leaves, not only is a decrease in chlorophyll content observed, but changes in secondary metabolites—such as free amino acids, catechins, carotenoids, and aroma compounds—are also detected. In albino tea cultivars, the degradation of L-theanine is reduced, resulting in greater accumulation; the total catechin content is lower; and the diversity of aroma compounds is diminished, which is unfavorable for the formation of desirable tea aromas [44,45,46,47]. Owing to differential gene expression, albino tea varieties generally present lower catechin levels and higher amino acid contents, contributing to a mellower and more refreshing taste. However, the biosynthesis of carotenoids, fatty acid derivatives, and aromatic terpenes is reduced, leading to weaker aroma characteristics.

### 2.3. The Impact of Genetic Factors on Aroma in Fresh Tea Leaves and the Underlying Metabolic Mechanisms

The unique aroma characteristics of different tea cultivars are attributed to significant variations in the composition and content of volatile metabolites, which result from differential expression of genes regulating the biosynthesis of volatile metabolites among different tea varieties. On the basis of their metabolic origins, tea volatiles can be classified into four categories: volatile terpenes (VTs), volatile fatty acid decomposition products (VFADs), volatile phenylpropanoid/benzenoid compounds (VPBs), and carotenoid-derived volatile compounds. The metabolic pathways of these compounds are illustrated in Figure 1c–f.

The differences in characteristic aroma compounds among different tea plant varieties are associated with the specific high expression of key biosynthetic genes. For example, in ‘Tieguanyin’, the high expression of COMT, CCoAOMT, and CAD genes promotes the accumulation of eugenol; in ‘Fuding Dabaicha’, the upregulation of LIS/NES increases the linalool content; however, in ‘Baihaozao’, the high expression of NES/GIS leads to the accumulation of trans-nerolidol; and in ‘Fujian Shuixian’, the high expression of ADH is positively correlated with the content of trans-3-hexen-1-yl acetate [48]. A previous study revealed that high-aroma oolong tea varieties (‘Jinmingzao’, ‘Jinguanyin’, and ‘Jinxuan’) contain higher levels of VTs than green tea varieties do, which is closely related to the high expression of the DCS gene (which regulates damascenone synthesis) in their leaves [49]. Moreover, in ‘Huangguanyin’ and ‘Jinguanyin’, the high expression of the ADH8 (alcohol dehydrogenase) and SPL2 (secretory phospholipase) genes may be key factors contributing to the accumulation of volatile fatty aldehydes (VFADs) [49].

### 2.4. The Impact of Genetic Factors on Caffeine in Fresh Tea Leaves and the Underlying Metabolic Mechanisms

The biosynthesis of caffeine, a purine alkaloid synthesized in young tea leaves, is initiated with xanthine as the precursor and is completed through three sequential methylation reactions (Figure 1h). Initially, 7-methylxanthine is catalyzed by 7-NMT from xanthine, followed by conversion to theobromine catalyzed by TS. Finally, theobromine is methylated to caffeine under the catalysis of TCS [50]. The caffeine content in tea is regulated primarily by the TCS gene via the following mechanisms: (1) allelic variations in TCS lead to alterations in enzyme activity, and (2) promoter variations in TCS affect its expression level. As the rate-limiting enzyme for caffeine synthesis, the activity of TCS directly determines the caffeine content in fresh tea leaves. Certain allelic variants can result in the inactivation of the enzyme, thereby blocking caffeine synthesis. For instance, ‘Nongdao Wild Tea’ has two TCS1 allelic variations, namely TCS1b with only theobromine synthetase activity and TCS1d with both theobromine and caffeine synthetase activities. Among them, the high expression of the TCS1b gene and the extremely low expression of the TCS1d gene are very likely the key reasons for the high theobromine and low caffeine contents in ‘Nongdao Wild Tea’ [51]. An investigation of caffeine-specific germplasms revealed that amino acid residue 269 in TCS1 plays a crucial role in TCS activity and substrate recognition. Compared with TCS1a, the Ser269Cys mutant of the TCS1a gene presented a 2.5-fold increase in caffeine synthase (CS) activity, and the TCS promoter, as well as certain genes that bind to the promoter, could directly or indirectly influence the expression of the TCS gene [51]. Li et al. reported that MYB184 can directly bind to and activate the TCS1 promoter, which might explain why the expression level of MYB184 in cocoa tea is significantly lower than that in cultivated tea trees [52]. The caffeine content in the ‘Jianghua Kucha’ variety was found to be significantly greater than that in other tea plant cultivars [15]. Further studies revealed that bHLH1 regulates caffeine biosynthesis through two distinct mechanisms. On the one hand, bHLH1 was observed to bind to the promoter of the TCS1 gene, thereby inhibiting its transcription and subsequently suppressing caffeine biosynthesis. On the other hand, the expression of bHLH1 was demonstrated to be repressed by miR1446a, leading to an increase in caffeine biosynthesis. On the other hand, the expression of bHLH1 was demonstrated to be repressed by miR1446a, leading to an enhancement in caffeine biosynthesis [15]. Additionally, the synthesis of caffeine or theobromine can also be influenced by certain regulatory factors through the modulation of TCS activity. It was discovered by researchers that the S40 factor, which is associated with aging regulation, negatively modulates TCS1 in the tea cultivar ‘Fuding Dabaicha’ and suppresses caffeine accumulation [53].

### 2.5. The Impact of Genetic Factors on L-Theanine in Fresh Tea Leaves and the Underlying Metabolic Mechanisms

The content of amino acids in fresh tea leaves varies among different tea plant cultivars due to differences in genetic material and metabolic pathways, the metabolic pathways are shown in Figure 1g. A total of 26 amino acids have been identified in tea leaves, including 6 nonprotein amino acids and 20 proteinogenic amino acids. Among these amino acids, L-theanine is the most abundant, accounting for more than 60% of the total free amino acids. L-theanine is synthesized in the roots of tea plants through the catalysis of L-theanine synthase, which facilitates the condensation of ethylamine and glutamic acid. It is then transported via vascular tissues to the leaves, where it accumulates. Subsequently, L-theanine is hydrolyzed by L-theanine hydrolase and participates in the metabolism of catechins and other amino acids. The synthesis of L-theanine is regulated by enzymes and genes involved in its biosynthesis, transport, and catabolism, while transcription factors further influence its expression by binding to these genes [54]. Liu et al. [55] identified seventeen enzyme genes related to L-theanine metabolism from the tea transcriptome database, including L-theanine synthase genes (*TS1*, *TS2*) and glutamine synthetase genes (*GS1*, *GS2*), among others. The transcript levels of most L-theanine metabolic enzyme-encoding genes in ‘Huangjinya’ were significantly greater than those in ‘Anjibaicha’ and ‘Yingshuang’, corresponding to the higher L-theanine content in ‘Huangjinya’. Similarly, it was reported that the elevated L-theanine content in the leaves of Huabai 1 was associated with high expression of the *GS1.2* and *GS2* genes [55].

Glutamate and ethylamine are important precursors in the synthesis of L-theanine, and different tea plant varieties influence the synthesis and accumulation of L-theanine through the differential expression of genes in these two metabolic pathways [54]. A study demonstrated that the enzymatic activities of GOGAT (CSS0007758) and GDH (CSS0034454), key proteases involved in L-glutamate synthesis, were significantly greater in the ‘ZB-1’ variety than in the ‘LYFS’ variety, leading to greater accumulation of L-glutamate in this cultivar [56]. High expression of the cadaverine synthase/alanine decarboxylase (AlaDC) gene is associated with increased accumulation of L-theanine [57,58]. Notably, L-theanine can be synthesized only in the root system of tea plants (Camellia sinensis). Therefore, the transport capacity of L-theanine from roots to fresh leaves is also a critical factor influencing its content in fresh leaves. The AAPs (amino acid permeases) subfamily is involved in the transport of various amino acids in the roots, phloem, and xylem of tea plants. As demonstrated by Li et al., heterologous expression in yeast mutants revealed that the tea plant amino acid permease AAP7.2 has L-theanine transport activity, and the overexpression of AAP7.2 in Arabidopsis was shown to increase the L-theanine content, while the accumulation of other amino acids was also significantly increased [59]. The transportation of L-theanine is differentially regulated by various AAP genes. High expression of AAP2 might inhibit the translocation of L-theanine from roots to shoots. Compared with ‘JB-2’ and ‘ZB-1’, the expression level of AAP2 was significantly upregulated in the tea cultivar ‘LYFS’, resulting in the lowest ratio of L-theanine content in leaves relative to that in roots in ‘LYFS’ [56]. Additionally, the degradation of L-theanine leads to a reduction in its content in fresh tea leaves. L-theanine is hydrolyzed by L-theanine hydrolase into glutamic acid and ethylamine in the leaves. Glutamic acid can be utilized for the synthesis of amino acids, proteins, chlorophyll, and other compounds, whereas ethylamine is oxidized by amine oxidase into acetaldehyde, which can be further used for the synthesis of the phloroglucinol nucleus of catechins [60]. The transcript level of the L-theanine hydrolase gene PDX2.1 in albino tea cultivars is lower than that in green tea cultivars, resulting in a greater L-theanine content [45]. GGT4 has been identified as a homologous protein of the L-theanine hydrolase GGT2. A sustained increase in the transcription factor MYB73 significantly upregulates the expression of GGT2 while simultaneously suppressing the expression of GGT4, thereby exerting a negative regulatory effect on L-theanine accumulation [61]. Genetic factors shape fresh tea quality, and, as in other crops, cultivation and environment modulate secondary metabolism [62,63]. These combined drivers redirect metabolite fluxes, ultimately altering leaf chemistry.

## 3. Influence of Environmental Factors on the Quality of Fresh Tea Leaves

The quality of tea plants is coregulated by the synergistic interaction between belowground and aboveground environments. In the belowground environment, soil pH, mineral elements, and microbial communities directly influence tea plant metabolism by modulating root development and nutrient uptake. In the aboveground environment, climatic factors such as temperature, light, and water primarily govern photosynthesis and the synthesis of secondary metabolites. These two components collectively regulate the synthesis and accumulation of natural metabolites in tea leaves through a complex interaction network, ultimately determining the quality characteristics of fresh leaves. These synergistic effects between aboveground and belowground ecosystems form the basis for the natural formation of flavor compounds in tea.

### 3.1. Influence of the Subsurface Ecological Environment of Tea Plantations on the Quality of Fresh Tea Leaves

#### 3.1.1. Soil Characteristics

As a nitrogen-preferring crop, the growth, development, and quality formation of tea plants are critically influenced by the three major elements: nitrogen, phosphorus, and potassium. Nitrogen significantly affects tea yield and quality, with appropriate application promoting chlorophyll synthesis and new shoot growth, increasing the contents of amino acids and caffeine, and influencing the synthesis and release of volatile compounds. Nitrogen deficiency increases the expression of enzyme-encoding genes (such as FLS, DFR, and ANS), promoting the synthesis of polyphenols while suppressing the expression of theanine synthesis genes (e.g., AlaDC and GDH), thereby impairing L-theanine production [64]. However, excessive nitrogen application can induce soil acidification and inhibit the synthesis of catechins and aromatic compounds [65]. Studies have also revealed that tea plants exhibit a selective preference for nitrogen sources and that NH_4_^+^ enhances amino acid accumulation, whereas NO_3_^−^ strengthens catechin synthesis, and that a balanced supply of both nutrients optimizes carbon and nitrogen metabolism, thereby regulating tea plant growth and the synthesis of secondary metabolites [64]. Nitrogen source morphology significantly alters the direction of nitrogen metabolism in tea plants. For example, NH_4_^+^ upregulates the expression of transporter genes such as NPF2.7, markedly increasing the contents of L-theanine, proline, and other amino acids. In contrast, NO_3_^−^ preferentially activates the expression of the ANS enzyme-encoding gene, promoting the synthesis of epicatechin. This divergence stems from the preferential assimilation of ammonium nitrogen in tea plants, which upregulates the expression of key enzyme-encoding genes (e.g., GSs) to directionally regulate amino acid biosynthesis [64,65,66]. Phosphorus (P) influences secondary metabolism by promoting energy conversion, with appropriate P application increasing the contents of tea polyphenol substances. The P-starvation-responsive transcription factor PHR1/2 directly activates ANR1 or indirectly regulates MYC5c, thereby affecting catechin synthesis [67]. Potassium (K) plays a critical role in photosynthesis and nitrogen metabolism, where optimal K fertilization has been demonstrated to increase the levels of tea polyphenols, amino acids, and volatile compounds [68]. In contrast, K deficiency has been shown to significantly reduce caffeine and theanine contents. Studies revealed that under K deficiency, the expression of N-methyltransferase (NMT) genes and tea caffeine synthase (TCS) genes was significantly downregulated, leading to a marked decrease in caffeine content; concurrently, the expression of theanine synthesis-related enzymes (TS, GS, GDH, and AIDA) was most notably altered compared with that in the control, resulting in a substantial reduction in L-theanine content [69]. Zhou and colleagues further demonstrated that increased soil K content could activate TSI, thereby promoting L-theanine accumulation [70]. Tea plants are characterized by aluminum (Al) accumulation, fluoride tolerance, and calcium (Ca) aversion. Low Al concentrations stimulate root growth and increase the accumulation of quality-related compounds, whereas excessive Al induces toxicity. Zhang et al. reported that under Al stress, key genes involved in catechin biosynthesis, such as 4-coumarate-CoA ligase 4, anthocyanidin reductase, flavanone 3-hydroxylase, leucoanthocyanidin reductase, and phenylalanine ammonia-lyase, were upregulated, contributing to Al detoxification [71]. Tea plants employ multiple mechanisms to tolerate fluoride (F), including cell wall chelation, vacuolar compartmentalization, Al/Ca-F complex formation in roots, and root secretion of organic acids for F chelation [72]. Within an appropriate range, F has been found to enhance catechin synthesis, whereas excessive F inhibits the formation of quality components, including free amino acids, tea polyphenols, caffeine, and aroma compounds. Calcium ions (Ca^2+^) have been shown to improve tea plant resistance to salt, cold, and drought stress. Excessive Ca^2+^ may increase the contents of amino acids, caffeine, and tea polyphenols in fresh leaves, where these secondary metabolites potentially act as antioxidants to mitigate Ca-induced stress [73]. Soil pH is recognized as a critical factor affecting tea plant growth and quality, with the optimal range identified as 4.0–5.5. Elevated pH has been associated with impaired nutrient uptake and chlorosis, whereas excessively low pH may trigger Al/Mn toxicity and activate heavy metal ion stress. Notably, nitrogen uptake efficiency peaks at pH 5.0, whereas amino acid and sugar contents remain relatively insensitive to pH variations [74]. In summary, these elements collectively shape tea quality traits by regulating the expression of key genes (e.g., GS1, TS1, and ANR1) and enzyme activities (e.g., PAL and CHS), forming an intricate metabolic network. Therefore, the precise management of soil nutrients and pH is essential for high-quality tea production.

#### 3.1.2. Soil Microorganisms

**Figure 2:**
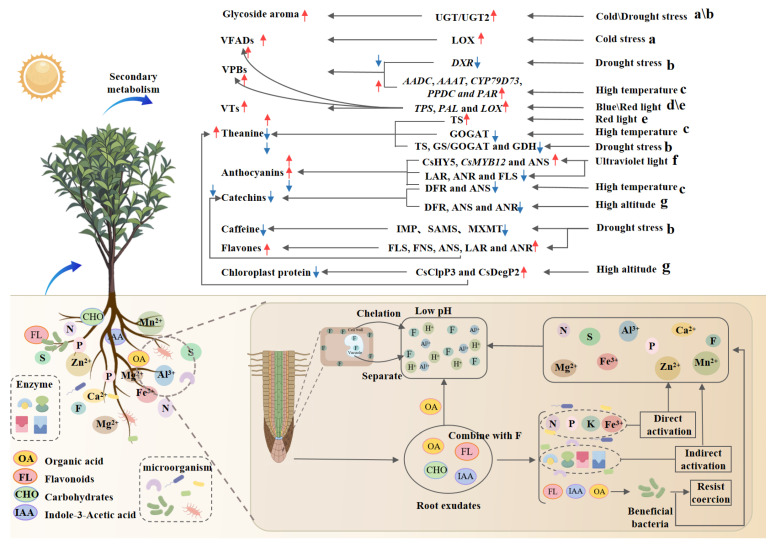
The influence of environmental conditions and microorganisms on the secondary metabolic synthesis of tea: (a) represents the pressure of low temperature; (b) stands for drought pressure; (c) stands for high temperature and pressure; (d–f) represent the influences of blue light, red light, and ultraviolet light, respectively; (g) represents the influence of altitude conditions.Red arrows indicate upregulated gene expression or enhanced enzyme activity, while blue arrows indicate downregulated gene expression or decreased enzyme activity.

Microorganisms are recognized as essential components of soil ecosystems, with tea garden soils exhibiting high microbial diversity and richness, comprising a wide range of fungi, bacteria, and viruses. The quality of tea is regulated by the secretion of antimicrobial substances and hormones and the promotion of nutrient cycling by soil microorganisms in tea gardens, which activate the immune system of tea plants, increase stress resistance, and promote the growth and development of tea leaves (Figure 2). Wei et al. [11] conducted a comparative analysis of the root microbiota between the high-L-theanine tea cultivar ‘Rougui’ and the low-L-theanine cultivar ‘Maoxie’. Their study identified a bacterial consortium that potentially modulates nitrogen metabolism, influencing leaf L-theanine levels. Based on these findings, they constructed a synthetic microbial community (SynCom). Upon inoculation, SynCom21 was shown to promote root growth in Arabidopsis by maintaining ammonium homeostasis, indicating that these microbes play a crucial role in governing nitrogen balance and subsequently determining theanine accumulation in tea plants. Moreover, the lead degradation mechanism mediated by the efflux transporter of the rhizobacterial strain AT31-1 in tea plants significantly reduces the lead content in tea leaves and improves quality, confirming the role of soil microorganisms in heavy metal pollution remediation [75]. The application of microbial organic fertilizers containing Bacillus megaterium, Bacillus colloid, and Bacillus subtilis has been demonstrated to significantly increase the contents of tea polyphenols, amino acids, and caffeine [76]. Additionally, soil microorganisms in tea plantations have been shown to enhance tea plant stress resistance and improve quality through multiple mechanisms. Under gray blight disease stress, tea plants were found to enrich beneficial microbial communities such as Trichoderma asperellum through phenolic acid secretions, which suppress pathogens via antimicrobial synthesis, immune induction, and niche competition, as reported by Wang et al. [77]. It has been confirmed that B. aryabhattai AB211 can synergistically alleviate stress through phosphate solubilization, siderophore synthesis, and IAA secretion [78]. Current research on microorganisms in tea gardens has revealed the effects of certain functional strains on the synthesis of theanine, heavy metal remediation, and disease inhibition. However, much of this research is limited to studies conducted on potted plants or simplified microbial communities. Furthermore, the mechanistic understanding has only reached the level of enzyme activity, and the relationship between the transcriptional signals of the rhizome and the metabolites in tea has not been systematically analyzed. There is a notable lack of multi-year and multi-site field experiments to verify the stability of microbial colonization. The next step is to integrate metagenomic tracking of strain dynamics and develop a ternary matching model of “microbial agent–tea tree variety–soil type” to customize bio-fertilizers for tea gardens, thereby enhancing both tea yield and quality.

### 3.2. Influence of Climatic Conditions on the Quality of Fresh Leaves

#### 3.2.1. Temperature

The physiological metabolism and quality formation of tea plants are significantly influenced by temperature. The optimal growth temperature for tea plants is 20–25 °C, with a critical low temperature of −6 °C. Under high-temperature conditions, the synthesis of secondary metabolites such as catechins, anthocyanins, and L-theanine in tea leaves is regulated by transcription factors, genes, and hormones (c in Figure 2). Studies have revealed that the heat shock transcription factor HSFA1b/2, which is activated by high temperatures, suppresses the jasmonic acid signaling pathway by binding to the promoter of JAZ6, thereby reducing catechin synthesis, whereas jasmonic acid, a defense hormone, can mitigate the inhibitory effect of high temperatures on catechin biosynthesis [12]. High temperatures also induce the upregulation of MYBL2a/b, which inhibits the expression of DFR and ANS, leading to reduced accumulation of catechins and anthocyanins [79]. It has been demonstrated that when temperatures reach 38 °C, L-theanine synthesis is decreased due to the downregulation of NADH-GOGAT, while high temperatures upregulate genes involved in 2-phenylethanol synthesis (AADC, AAAT, CYP79D73, PPDC, and PAR), promoting the accumulation of volatile phenyl compounds [80,81]. Low-temperature stress induces the accumulation of cold-resistant substances such as amino acids, carbohydrates, ascorbic acid, and flavonoids (kaempferol, quercetin, myricetin, and catechins). Short-term low temperatures also activate the expression of LOX enzyme genes (LOX1, LOX6, and LOX7), promoting the synthesis of aroma compounds [82]. In addition to temperature magnitude, diurnal temperature variation significantly affects tea plant metabolism. Research indicates that a 10 °C diurnal temperature difference is most conducive to amino acid accumulation, which is attributed to the high expression levels of the L-theanine synthase gene (TS3) and glutamate synthase gene (GOGAT) under such conditions [83]. In a study on the ‘Fuyun 6’ tea cultivar subjected to four diurnal temperature treatments, the contents of free amino acids, caffeine, and hexanal increased significantly with greater diurnal temperature differences, whereas the total flavonoid content, phenol-to-amino acid ratio, and leaf alcohol content exhibited opposite trends [84]. However, other studies have reported that caffeine and free amino acid contents initially increase but then decrease with increasing diurnal temperature, whereas catechin and tea polyphenol contents exhibit the opposite pattern [85]. These findings suggest that, within a certain range, an increased diurnal temperature difference helps reduce the phenol-to-amino acid ratio, enhancing the freshness of tea, whereas the specific temperature difference yielding the highest phenol-to-amino acid ratio is determined by the tea cultivar.

#### 3.2.2. Light

Light regulation plays a crucial role in modulating secondary metabolism and quality in tea plants. Moderate light exposure has been shown to reduce the phenol-to-amino acid ratio and increase freshness, whereas intense light (particularly UV-B) has been demonstrated to upregulate genes such as FLS, thereby increasing flavonol glycoside accumulation and increasing bitterness and astringency while simultaneously suppressing ECGT activity, leading to reduced synthesis of esterified catechins [86]. Additionally, light quality has been reported to significantly influence the metabolic profile of tea leaves [87]. Blue light (200 μmol m^−2^ s^−1^) has been found to promote anthocyanin and catechin synthesis, but its high intensity has been shown to inhibit catechin and fatty acid metabolism [87]. Red light has been shown to upregulate GSTs genes, enriching umami amino acids, while downregulating PAL and LAR enzyme genes, thereby suppressing flavonoid metabolism [88]. Ultraviolet light has been demonstrated to enhance bitterness and astringency in tea leaves through the expression of HY5 and MYB12 [89]. Additionally, by reducing the expression levels of LAR, ANR, and FLS enzyme-encoding genes while increasing ANS enzyme-encoding gene expression, UV light has been shown to promote anthocyanin synthesis, explaining the deeper purple coloration observed in purple tea varieties under high summer UV exposure [88,89]. Fu et al. (2015) revealed that blue/red light significantly enhances the synthesis of aroma compounds, including terpenes, phenylpropanoids, and fatty acid derivatives, through the upregulation of TPS, PAL, and LOX gene expression (d,e in Figure 2) [90]. Currently, light regulation technology has been applied in tea processing, such as withering upregulates the genes for the synthesis of phenylalanine and tryptophan, leading to an increase in amino acid content [91]. While shading cultivation has been widely adopted, the potential for light quality regulation remains underexplored. Future research should focus on optimizing light conditions according to seasonal and growth-stage requirements to precisely modulate quality-related components.

#### 3.2.3. Moisture

As a hygrophilous but waterlogging-sensitive plant, tea has a physiological metabolism, and tea quality is affected by both excessive and insufficient water supply. Generally, moderate short-term drought stress has been shown to activate the antioxidant defense system of tea plants, significantly promoting the accumulation of glycosylated flavonoids while simultaneously inhibiting the biosynthesis of catechins, caffeine, L-theanine, and certain free amino acids. However, prolonged or severe drought has been reported to disrupt normal metabolic processes in tea plants [92,93,94]. It was found that under short-term drought conditions, the expression of key genes such as PAL, C4H, 4CL, CHS, and DFR was upregulated, enhancing reactive oxygen species (ROS) scavenging capacity [92]. However, prolonged or severe drought has been demonstrated to impair normal metabolism, ultimately leading to a significant decrease in catechin content. Similarly, Wang et al. (2016) found that drought stress significantly suppressed the expression of key genes involved in L-theanine synthesis (GS, GOGAT, and GDH) and caffeine biosynthesis (IMP, SAMS, and MXMT) while increasing the expression of flavonoid synthase genes (FLS and FNS) [92]. Consequently, the contents of catechins, caffeine, and L-theanine significantly decreased, whereas the total flavonoid content increased. Consistent with these findings, Li et al. (2021) revealed that moderate drought stress significantly inhibited the expression of TS (a key gene in L-theanine synthesis) and catechin-related genes (ANS, LAR, and ANR), leading to decreased levels of these compounds [94]. Moreover, the expression of PKSB (CSA026957, involved in naringenin chalcone synthesis) and FLS (flavonol synthase) was upregulated, promoting the accumulation of glycosylated flavonoids and isoflavonoids while reducing the catechin and anthocyanin contents [94]. Additionally, drought stress was found to upregulate genes related to terpenoid and fatty acid biosynthesis, as well as phenylalanine metabolism, resulting in increased accumulation of aromatic compounds such as linalool, geraniol, and phenylethanal, thereby enhancing tea aroma [95]. In addition, the combined treatment of drought and shading may activate the JA and ABA pathways during the pre-harvest fresh leaves and post-harvest wilting process, thereby enhancing the accumulation of esters and alcohols in black tea and white tea [96]. Notably, certain glycosyltransferases (e.g., UGT2) exhibit increased expression under drought conditions; these enzymes catalyze the glycosylation of aroma precursors such as perillyl alcohol and HMF, promoting the synthesis of O-glucosidic aroma compounds, which improves tea aroma quality and enhances drought resistance [97].

#### 3.2.4. Altitude

The influence of altitude, another influencing factor affecting the composition and content of secondary metabolites in fresh tea leaves, is attributed primarily to altitudinal gradient variations in environmental factors such as temperature, light, and water. Research has demonstrated that high-altitude environments are more conducive to the formation of amino acids and aromatic substances, whereas low-altitude conditions significantly promote the biosynthesis of catechins and caffeine [10,98]. With increasing altitude, the high expression of the chloroplast protease complex subunit 3 gene (ClpP3) and serine protease 2 gene (DegP2) leads to enhanced degradation of chloroplast proteins, thereby facilitating the accumulation of L-theanine precursors [10]. Simultaneously, the genes (DFR, ANS, and ANR) involved in catechin synthesis are significantly downregulated, while the transcription factor MYB4, which inhibits the expression of ANR and LAR, is markedly upregulated, resulting in reduced catechin content [10]. Additionally, in fresh leaves, the expression levels of PAL and the aromatic amino acid transaminase gene (AAAT1) are upregulated at high altitudes, whereas the expression of phenylacetaldehyde reductase (PAR2) and phenylpyruvate decarboxylase (PPDC2) is downregulated, leading to increased L-phenylalanine content but decreased phenylacetaldehyde and 2-phenylethanol content, thereby altering tea aroma [10]. However, Ran et al. (2023) revealed that as altitude increases, amino acid content gradually rises, while catechin content initially decreases and then increases [99]. The content of flavonoids, alcohols, and glycosides shows no significant variation across different altitudes, whereas volatile components such as aldehydes, ketones, and methyl salicylate slowly increase with elevation, which may be related to differences in soil properties, climate, and latitude [100]. A certain degree of altitude is beneficial for reducing the phenol-to-amino acid ratio, enhancing tea aroma, and ultimately improving tea quality.

#### 3.2.5. Season and Developmental Stage

Seasonal profiling reveals a rhythmic quality paradigm characterized by ‘spring freshness and summer bitterness.’ Theanine levels peak in spring, while catechins reach their highest concentration in summer; in contrast, caffeine levels remain consistent throughout the seasons. Additionally, the winter chill enhances aroma [101,102]. These seasonal rhythms are primarily driven by irradiance-mediated photosynthesis, which modulates both transcription factors (TFs) and structural genes involved in metabolite pathways. Gong et al. integrated transcriptomics and metabolomics from one-bud-one-leaf samples of ‘CheYunZhong’ collected in spring, summer, and autumn, documenting a positive correlation between the most abundant catechin, EGCG, and DFR (CSA003950). Furthermore, SAMDC (CSA029628), which is closely linked to TFs, tracked the seasonal abundance of theanine [101]. Zheng et al. further demonstrated that winter cold upregulates UDP-glycosyltransferase genes, enhancing TV synthesis and ultimately increasing aroma intensity [102].

Leaf ontogeny imposes a decisive chemical filter that significantly influences metabolomic composition, architecture, and titre as the shoot matures. Theanine levels decline linearly with leaf age [103], while caffeine, theobromine, L-theanine, and catechins reach their nadir in older leaves [54,104]. Xu et al. [56] demonstrated that simple catechins (C, EC, EGC) accumulate with increasing maturity, whereas esterified forms (ECG, EGCG) and total catechins exhibit an opposite trajectory; caffeine and gallic acid decline simultaneously. The abundance of monoterpenes decreases with leaf age, while fatty-acid-derived volatiles display a contrasting gradient [69]. Wu et al. [105] reported a maturity-dependent increase in polyunsaturated fatty acids at the expense of saturated and monounsaturated species, thereby imprinting distinct aroma signatures on processed tea. Non-terpenoid profiling revealed a continuous decline in total nitrogen alongside a concomitant starch accumulation as leaves mature, indicating a developmental shift in source-sink relations that reconfigures primary and secondary metabolism [54]. Consequently, harvest timing emerges as a critical determinant of leaf quality, influencing the secondary metabolite repertoire. Yao et al. [69] established that the senescence-associated gene CsS40 negatively regulates TCS1, leading to a reduction in caffeine synthesis as CsS40 expression increases with aging. Liu et al. [106] demonstrated that the distribution of theanine across three cultivars (‘Yingshuang’, ‘Anjibaicha’, and ‘Huangjinya’) is conserved, linking its decline to rising transcript levels of CsTS1 and CsGOGAT-Fe, and decreasing levels of CsTS2, CsGS1, and CsGDH2. Transcriptomic surveys conducted by Ajay Kumar et al. [107] across these three cultivars further indicated that developmental stage exerts a stronger influence than seasonal fluctuations on the expression of core genes involved in catechin biosynthesis (F3ʹH, F3ʹ5ʹH, FLS, DFR, LAR, ANR, and ANS) and caffeine metabolism (MXMT, SAMS, TCS, and XDH).

Collectively, the quality of tea is governed by a complex molecular interplay among seasonal factors and ontogeny, ultimately modulated by temperature, light, and the carbon-nitrogen balance. Clarifying these mechanisms provides a solid framework for determining the optimal picking window to enhance the quality of fresh leaves.

## 4. Influence of Management Measures on the Quality of Fresh Tea Leaves

Based on in-depth research on the metabolic patterns of secondary metabolites in tea plants and their interactions with the environment, scholars have adopted various management measures, such as changing cultivation methods, fertilization, and pruning, to optimize the growth and development patterns of tea plants and further regulate the quality of fresh tea leaves.

### 4.1. Cultivation Pattern

**Figure 3:**
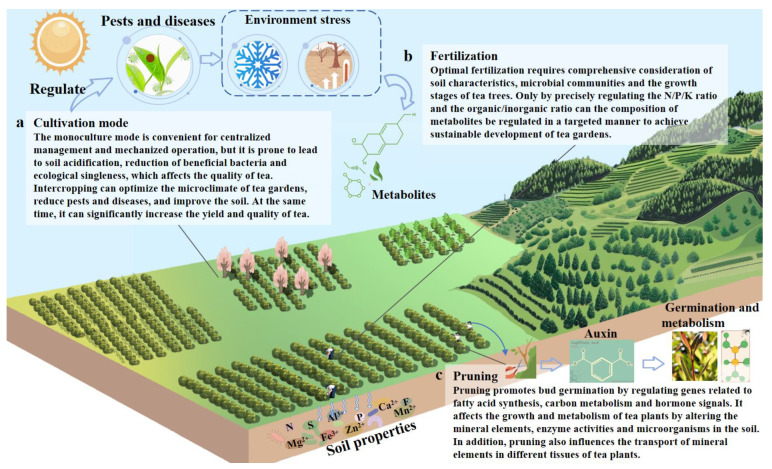
The impact of management on tea quality: (**a**–**c**) represent the effects of cultivation patterns, fertilization, and pruning on the growth, metabolism, and quality of tea plants, respectively.

Tea plants are perennial woody plants, and their cultivation methods include monoculture, intercropping, and relay intercropping. The monoculture mode facilitates centralized management and mechanized operation but is prone to soil acidification, a reduction in beneficial microorganisms, and ecological simplification, which negatively affect tea quality [100]. Therefore, intercropping and relay-cropping systems in tea plantations have been increasingly adopted in recent years, as they not only optimize the microclimate, reduce pests and diseases, and improve soil quality but also significantly increase tea yield and quality (Figure 3a). Studies have demonstrated that intercropping can improve soil enzyme activity, influence the structure and function of microbial communities, and promote the recycling of nutrient elements [108]. In addition, the intercropping mode has been proven effective in regulating tea plantation pests and diseases. There are two reasons for this. Firstly, the number of natural enemies (such as parasitic wasps and predatory insects) has increased, significantly suppressing the population growth of major pests like leafhoppers, aphids, and ephedra moths. Secondly, when plants such as cinnamomum cassia, alfalfa, mint, motherwort, and cornus officinalis are intercropped, their released volatile secondary metabolites exhibit significant repellent effects on pests, thereby reducing pest incidence [108]. Interestingly, some intercropped plants can influence tea plant growth and tea quality through allelopathy. Gao et al. (2024) found that allelochemicals such as benzaldehyde, α-farnesene, and methylbenzene from flowering cherry trees promote the formation of floral aroma compounds in tea leaves, including cis-jasmone, linalool, and nonanal, ultimately enhancing tea aroma [109]. Wang et al. (2024) revealed that chestnut-tea intercropping activates the *WRKY48* gene through photoreception and allelopathy, subsequently upregulating cold resistance genes (*CBF*, *DREB*) and pest resistance genes (*ChiA*), and through interactions with transcription factors such as CBF, ERF, MYC, and MYB, the resistance of tea plants to biotic and abiotic stresses is enhanced [110].

Furthermore, intercropping can improve the climatic conditions of tea plantations by modulating humidity and light intensity, thereby influencing tea quality. However, it should be noted that while numerous studies have confirmed the significant benefits of intercropping in improving soil conditions, controlling pests and diseases, and enhancing tea quality, some studies have also identified potential negative effects associated with specific intercropped plants. For example, in tea plantations intercropped with walnut trees, a decline in soil microbial abundance has been observed, along with increased flavonoid, polyphenol, and alkaloid contents but reduced amino acid levels in tea leaves, leading to a more bitter and astringent tea infusion [111]. Therefore, further research is needed on intercropped plants that may adversely affect tea quality.

### 4.2. Fertilization

Fertilization affects the absorption of nutrients by tea plants through the regulation of soil nutrients, thereby modulating the synthesis and metabolism of secondary metabolites in tea leaves (Figure 3b). The balanced application of N, P, and K fertilizers is crucial for tea growth and quality. Nitrogen is the primary limiting factor, significantly influencing the contents of amino acids, tea polyphenols, and caffeine [112]. Research has found that nitrogen deficiency reduces the synthesis of L-theanine while promoting the accumulation of catechins, but long-term nitrogen application leads to soil acidification and a decline in available phosphorus [112,113]. Yang et al. (2020) found that nitrogen fertilization upregulates the expression of L-theanine synthesis-related genes, including DHQ/SDH, AlaDC, AspAT, PAT, and SHMT, resulting in increased accumulation of L-theanine and other amino acids [102]. Phosphorus and potassium fertilizers promote the accumulation of catechins and carbohydrates but inhibit amino acid synthesis. For instance, Wei et al. (2022) demonstrated that P and K fertilizers enhance malate metabolism in tea buds, inducing the redistribution of photosynthetic products and carbohydrates toward the catechin pathway [112]. In addition to nutrient ratios, the use of organic and inorganic fertilizers has been a focus of research. Organic fertilizers play a significant role in improving microbial communities and alleviating soil acidification, while inorganic fertilizers ensure nutrient supply [112]. The synergistic application of these compounds increases the contents of catechins, amino acids, and other metabolites. According to Raza et al. (2023), the combined application of inorganic and organic fertilizers upregulates the expression of DFR2, ANS3, and ANR2 genes, promoting catechin synthesis [114]. Beyond conventional organic and inorganic fertilizers, microbial organic fertilizers, such as Bacillus spp., have been shown to significantly (*p* < 0.05) increase the activities of soil enzymes, including leucine aminopeptidase, β-glucosidase, β-N-acetylglucosamine, acid phosphatase, β-cellobiosidase, and β-xylanase. This enhancement improves nutrient utilization efficiency and leads to a greater accumulation of tea polyphenols, amino acids, and caffeine in fresh leaves [76]. Therefore, optimized fertilization requires comprehensive consideration of soil properties, microbial communities, and tea plant growth stages. Only through precise regulation of the N/P/K ratios and organic/inorganic combinations can the metabolic composition be directionally controlled, enabling sustainable tea plantation management.

### 4.3. Pruning

Pruning is recognized as a crucial measure in tea cultivation management and is employed to delay tea plant senescence, promote growth, and increase yield (Figure 3c). The sprouting of buds is facilitated through the regulation of genes associated with fatty acid synthesis, carbon metabolism, and hormone signaling by pruning, while phenylpropanoid metabolism and the TCA cycle are activated to strengthen nutrient absorption by lateral roots [115,116]. It was demonstrated that genes related to auxin, vitamin synthesis, and signaling are upregulated by pruning, thereby promoting the growth and development of tea buds [117]. An increase in the contents of the hormones L-phenylalanine and iPAMP was observed due to pruning, which enhances stress resistance and stimulates lateral bud sprouting [115]. Simultaneously, key pathways including fatty acid synthesis, carbon–nitrogen metabolism, and protein processing are regulated at the genetic level by pruning, synergistically advancing tea plant growth [116]. Furthermore, the synthesis of secondary metabolites in tea leaves is influenced by pruning. Compared with those in unpruned tea plants, genes involved in the biosynthesis of catechins, flavonoids, and caffeine were significantly upregulated by pruning, leading to the accumulation of compounds such as kaempferol, myricetin, quercetin glycosides, and caffeine, thereby intensifying the astringency and bitterness of tea infusions [116]. To preserve mellow taste and pronounced aroma, traditional ancient tea gardens often avoid pruning, as it results in a reduction in flavor quality. This practice significantly decreases the content of the bitter-tasting EGCG by downregulating catechin synthesis genes such as CHS and F3H, while genes related to chlorophyll metabolism and tyrosine metabolism are upregulated, promoting the accumulation of free amino acids, chlorophyll, and aromatic substances, thereby forming a refreshing taste and distinctive aroma [116,118]. Additionally, soil mineral element contents, enzyme activities, and microbial communities are altered by pruning, subsequently affecting tea plant growth and metabolism. The increased activities of soil catalase, acid phosphatase, and sucrase have been reported to be due to pruning, which improves organic matter decomposition efficiency and available phosphorus content, while the abundance of rhizosphere soil microbes such as Haliangium, Acidicaldus, and Reyranella is modified, thereby promoting tea plant growth and yield [116,119]. Interestingly, the translocation of mineral elements within different tissues of tea plants is also affected by pruning. A reduction in the concentrations of mobile elements such as N, P, K, and Mg in tea leaves was observed by Liu et al. (2022) [120], whereas the concentrations of immobile elements such as Ca, Al, Mn, B, and Co increased.

## 5. Genetic, Environmental, and Management Factors Interact with Each Other

The aforementioned research indicates that the secondary metabolites of tea plants are influenced by genetic, environmental, and management factors (Table S1); however, they are not determined by a single factor alone. Instead, these metabolites result from the coupled interaction among genetic potential, environmental influences, and agronomic practices. The genetic background establishes the baseline allocation of metabolic pathways. For instance, two strains from the same hybrid combination, due to variations in the expression of key structural genes, direct the carbon–nitrogen flow towards catechins (‘Zhongcha 108’, which exhibits high expression of PAL/F3H/LAR) or theanine–caffeine (‘Zhongming 7’, which shows high expression of GOGAT/GS/TCS) [121]. Environmental factors exert selective pressure by altering the physical and chemical properties of the soil and the structure of microbial communities. An increase in altitude reduces the abundance of total phosphorus (TP), total potassium (TK), and functional bacteria such as Fibrobacteres and Stachybotrys in the soil, while simultaneously inhibiting the contents of catechins, theanine, and caffeine [122]. Furthermore, combined cold and drought stress triggers overlapping transcriptional responses, delays leaf senescence, and induces a 31.5% non-additive differential gene reconfiguration in secondary metabolism [123]. Agronomic interventions act as tunable modulators that either amplify or buffer environmental inputs. Medium-density tea–forest intercropping (T2 + M) lowered canopy temperature by 5.5 °C and raised relative humidity by 6.6%, while elevating plant-available N, P and K in the 0–100 cm soil horizon by 41–230%. These edaphic–microclimate adjustments translated into 50–212% higher free amino acids and 92–242% more catechins relative to monoculture controls [124]. Likewise, controlled pre-harvest drought superimposed on 80% shade reduced leaf temperature yet increased ambient moisture, triggering jasmonate- and abscisate-mediated signalling (AOX×65, NCED×300) that drove the de novo synthesis of eighteen key aroma compounds—including benzyl alcohol, linalool and β-ionone together with marked theaflavin accrual, thereby rescuing summer-tea quality under heat stress [96]. Collectively, genotype furnishes the metabolic blueprint, the environment imposes selective pressure, and management refines resource availability and signalling thresholds; their integrated output defines the ultimate secondary metabolism of tea.

## 6. Conclusions

This review provides valuable insights into the influencing factors and regulatory mechanisms underlying the biosynthesis of secondary metabolites such as amino acids, caffeine, tea polyphenols, and volatile compounds in fresh tea leaves. In terms of secondary metabolism, many studies have revealed that secondary metabolism varies across varieties and different cultivation environments, highlighting the influence of genetic factors and local environmental conditions. Genetic diversity among tea plant varieties leads to significant variations in metabolic pathways, influencing key flavor- and health-related compounds. For example, allelic variations in the TCS and ANR genes determine caffeine and catechin profiles, whereas differential expression of AAPs, TS, and GGTs modulates L-theanine accumulation. Additionally, purple and albino tea cultivars present distinct metabolic patterns due to anthocyanin and chlorophyll-related gene expression, further diversifying tea quality traits. Environmental factors, including soil properties, microbial communities, temperature, light, and altitude, play crucial roles in shaping tea leaf quality. Soil nutrient availability (N, P, K, Al, and F) and pH influence nitrogen metabolism and enzymatic activity, whereas microbial interactions increase nutrient cycling and stress resistance. Climatic conditions, such as diurnal temperature fluctuations, UV exposure, and drought stress, alter the expression of genes involved in flavonoid, terpenoid, and amino acid biosynthesis. High-altitude cultivation, for example, promotes amino acid accumulation while reducing catechin content, contributing to a mellower taste profile. Cultivation management strategies, including intercropping, fertilization, and pruning, offer practical approaches to optimize tea quality. Intercropping with specific plants enhances soil health, pest resistance, and aroma compound synthesis, although improper pairings may negatively impact flavor. Balanced fertilization (organic/inorganic blends, microbial amendments) improves nutrient uptake and metabolic efficiency, whereas pruning regulates hormonal signaling and secondary metabolite production, albeit with potential trade-offs in astringency and aroma intensity.

In summary, this comprehensive review enhances our understanding of the influence and regulatory molecular mechanisms of the quality components of fresh leaves, offering valuable information for the development of effective cultivation and management strategies for high-quality tea. This study contributes to a broader understanding of the interrelationship between tea plants and environmental factors and emphasizes the need for continuous research to address emerging challenges in the context of the demands of tea production and changing environmental conditions.

## Figures and Tables

**Table 1:** Specific germplasm resource indicators.

Secondary Metabolites		Total Content	Varieties	References
Purine alkaloid	Caffeine	≥5.0%	‘Jianghua Strong Tea’; ‘Preferred No. 1’; ‘Preferred No. 6’; ‘Preferred No. 11’; ‘Preferred No. 13’; ‘Nanquan No. 1’; ‘Nanquan No. 2’; ‘Sage’; ‘Water Beetle’; ‘CMC24’, ‘DT20’; ‘ZP03’; ‘Shangyunbao Black Tea’	[16,17]
		≤1.5%	C. crassicolumna var. Multiplex; ‘Yuanbaoshancha’; ‘Huangshan Ku Cha’; ‘Hongyacha’; ‘Jin Chang Da Shu Cha’; ‘Ba Da Da Shu Cha’; ‘CafLess1’; ‘CafLess2’	[18,19]
	Theobromine	≥1.0%	C. Ptilophylla; C. irrawadiensis; C. gymnogyna; ‘Hongyacha’; ‘Yuanbaoshancha’; Camellia yungkiangensis; Camellia costata	[19]
	Theacrine	≥2.5%	‘Baiyacha’; ‘Bald House Tea’; ‘DT06’; ‘DT07’; ‘YX16’; ‘YX11’	[16,19]
Polyphenols		≥25.0%	‘Busyga Daishan Tea’; ‘Black Longleaf Tea’; ‘Youanbei White Toothed Tea’; ‘Big Black Tea’; ‘Yellow Bud Tea’; ‘Mengwen Tea’	[17]
Catechins and their derivatives	Catechin	≥20.0%	C. sinensis cv. ‘Fudingdabai’; ‘Shangyunbao Red Tea’	[17]
	EGCG	≥13.0%	‘Mengshan No. 11’; ‘Huaqiu No. 1’; ‘Huishan Yellow Large-Leaf Tea’; ‘Daping Large-Leaf Tea’; ‘Baotai Red Tea’; ‘Guangxi Hengxian Small Variety’; ‘Yichang Large-Leaf Variety’; ‘Xixiang Dahai No. 12’	[20]
	EGCG3”Me	≥1.0%	TRICAAS-1; TRICAAS-2; TRICAAS-3; TRICAAS-4; s ‘Jinmudan’; d ‘Jinguanyin’	[21,22]
Amino acid	Total amino acids	≥5.0%	‘Baojing Golden Tea No. 1’; ‘Golden Tea No. 2’; ‘Anji White Tea’; ‘Lingyun No. 1’; ‘Lingyun No. 2’; ‘Lingyun No. 7’; ‘Lingyun No. 10’	[20]
	Theanine	≥3.0%	‘Anji White Tea’; ‘Golden Bud’; ‘White Leaf No. 1’; ‘Little Snow Bud’; ‘Thousand-Year Snow’; ‘Lingyun No. 1’; ‘Lingyun No. 2’; ‘Anji White Tea’; ‘Simei Snow Bud’; ‘White Leaf No. 1’; ‘Zhonghuang No.1’; ‘Zhongbai No. 4’; ‘Jingbai No. 1’	[20,23]

## Data Availability

No data were used for the research described in the article.

## Supplementary Materials

The following supporting information can be downloaded at: https://www.mdpi.com/article/10.3390/foods14183268/s1, Table S1: Summary of the influencing factors of fresh tea quality and their effects; Table S2: Research details of cited literature.

## Abbreviations

The following abbreviations are used in this manuscript:

PAL	Phenylalanine ammonia-lyase
C4H	Cinnamate 4-hydroxylase
4CL	4-coumarate-CoA ligase
CHS	Chalcone synthase
CHI	Chalcone Isomerase
F3′5′H	Flavonoid-3′5′-hydroxylase
F3′H	Flavonoid 3′-hydroxylase
F3H	Flavonoid 3-hydroxylase
FLS	Flavonol synthase
DFR	Dihydroflavonol-4-reductase
ANS	Anthocyanin synthase
UFGT	Flavonoid-3-Oglucosyltransferase
ANR	Anthocyanidin reductase
LAR	Leucoanthocyanidin Reductase
UGGT	Galloyl-1-O-β-d-glucosyltransferase
ECGT	(epicatechin:1-O-galloyl-β-D-glucose-O-galloyltransferase
CsFAOMT1	3″-O methyltransferase
CsFAOMT2	4″-O methyltransferase
GluRS	Glutamine tRNA synthetase
HEMA	Glutamyl-tRNA reductase
HEML	Glutamate-1-Semialdehyde 2,1-Aminotransferase
HEMB	5-Aminolevulinic Acid Dehydratase
HEMC	Porphobilinogen deaminase
HEMD	Uroporphyrinogen III Synthase
HEME	Uroporphyrinogen decarboxylase
HEMF	Coproporphyrinogen-III oxidase
HEMG	Protoporphyrinogen oxidase
CHLH/I/D	Magnesium Chelatase H/I/D Subunit
CHLM	Magnesium protoporphyrin IX methyltransferase
DVR	Divinyl reductase
POR	Protochlorophyllide Oxidoreductase
CAO	Chlorophyllide a oxygenase
CHLG	Chlorophyll Synthase
CLH	Chlorophyll synthetase
NOL	Chlorophyll b reductase
SGR	Mg-Dechelatase
AADC	Aromatic Amino Acid Decarboxylase
PAAS	Phenylacetal-dehyde synthase
PAR	Phenylacetaldehyde reductase
AAAT	Aromatic amino acidaminotransferase
PAL	Phenylalanineammonia-lyase
PPAR	Phenylpyruvic acid reductase
CsAlaDC	Alanine decarboxylase
GS/GOGAT	Glutamine synthetase
GDH	Glutamate dehydrogenase
TS	Theanine synthase
ThYD	Theanine hydrolase
LOX	Lipoxygenases
HPL	Hydroperoxide lyase
ADH	Alcohol dehydrogenase
AOS	Acryloyl-coenzyme A synthase
AOC	Aldehyde oxidase cyclase
OPR	12-Oxo-phytodienoate reductase
JMT	Jasmonic acid carboxyl methyltransferase
CCD	Carotenoid cleavage dioxygenase
GES	Geraniol synthase
SLIS	(S)-Linalool synthase
RLIS	(R)-Linalool synthase
AMPD	Adenosine monophosphate deaminase
IMPDH	Inosine monophosphate deaminase
5′-Nase	5′-Nucleotidase
7-NMT/MXMT	7-Methylxanthine methyltransferase
N-MeNase	N-Methyl nucleosidase
TS	Theobromine synthase
TCS	Theacrine synthase
SCPL	Serine carboxypeptidase-like proteins
MYB	Myeloblastosis
bHLH	basic helix-loop-helix
VPBs	Volatile phenylpropanoid/benzenoids
VFADs	Volatile fatty acid decompositions
VTs	Volatile terpenes
COMT	Caffeic acid O-methyltransferase
CCoCOMT	Caffeoyl-CoA O-methyltransferaseevm
CAD	Alcoholdehydrogenase
PPDC	Phenylpyruvate decarboxylase
DHQ/SDH	3-dehydroquinic acid/succinate dehydrogenase
AspAT	Aspartate aminotransferase
PAT	Phosphinothricin acetyhransferase
AAPs	Amino acid permease
AIDA	Alanine decarboxylase
PKSB	Polyketidesynthases B
SHMT	Serine hydroxymethyltransferase
TCA	Citrate cycle
iPAMP	N-6-iso-pentenyladenosine-5′-monophosphate
HPLC	High Performance Liquid Chromatography
LC-MS	Liquid Chromatography-Mass Spectrometry
GC-MS	Gas Chromatography-Mass Spectrometry
qPCR	Quantitative Real-time Polymerase Chain Reaction
PCR-RFLP	Polymerase Chain Reaction-Restriction Fragment Length Polymorphism
qRT-PCR	Quantitative Real-time Reverse Transcription Polymerase Chain Reaction
SMRT	Single-molecule real-time
DNBSEQ	DNA Nanoball Sequencing
RT-PCR	Reverse Transcription Polymerase Chain Reaction
UPLC-MS/MS	Ultra Performance Liquid Chromatography-Tandem Mass Spectrometry
UHPLC-Orbitrap-MS/MS	Ultra-High Performance Liquid Chromatography Combined with Orbitrap Mass Spectrometry
RP-HPLC	Reversed Phase High Performance Liquid Chromatography
