# Supplementary material for: Influencing Factors and Regulatory Mechanisms of Fresh Tea Leaf Quality: A Review
Source: Foods. 2025 Sep 20;14(18):3268. doi: 10.3390/foods14183268 (PMC12469931; doi:10.3390/foods14183268)
Supplement: Supplementary file 1 [file foods-14-03268-s001.zip › foods-3868117-supplementary.pdf]

Table S1. Summary of the influencing factors of fresh tea quality and their effects

The main categories of influencing factors	Specific Category	Specific factors	Main affected quality attributes	References
Genetic factors	Tea Plant Variety	Tea plant variety	Catechins, nongalloylated/galloylated catechins, anthocyanins, aroma volatiles (VTs, VFADs, VPBs, VDFCs), caffeine and L-theanine	[8,15,27,28,34,35,42,45,48,49,51-53,56,69]
Environmental factors	Underground Environment	Soil pH	High pH → amino acids↓, leaf yellowing	[73]
		Nitrogen form (NH_4^+/NO_3^-)	NH_4^+↑ → amino acids, caffeine↑; NO_3^- → catechins↑	[64-66]
		Phosphorus (P) supply	Polyphenols, aroma↑	[67]
		Potassium (K) sufficiency	Caffeine, theanine, VTs↑	[68,70]
		A1	Catechins, flavonoids↑	[71,76]
		Microbial organic fertilizer (Bacillus complex)	Tea polyphenols, amino acids, caffeine↑	[11,74-77]
	Above-ground Climate	38 °C heat stress	Catechins, anthocyanins, theanine↓; 2-phenylethanol↑	[12,78-80]
		Cold stress	VFADs↑	[81]
		day–night temperature difference ↑	Amino acids, caffeine↑, total flavonoids↓	[84]
		Blue light 200 μmol m^{-2} s^{-1}	Anthocyanins, VTs, VPBs↑; ester-type catechins↓	[86,89]
		Red light	Amino acid, VTs, VPBs↑	[87,89]
		High light / UV-B (20 μW cm–2, 8 h/day)	Flavonol glycosides↑; galloylated catechins↓	[85,87,88]
		Short-term drought	aroma (linalool, geraniol)↑; catechins, caffeine, theanine↓	[93,95,96]
		Altitude ↑ (600→1200 m)	Amino acids↑, catechins↓; aroma (aldehydes, ketones, methyl salicylate)↑	[10,98,99]
	Season	Spring	Theanine content highest	[100]
		Summer	Catechin content highest	[100]
		Autumn	Catechins↓	[100]
		Winter	Theanine rebounds, TVs↑	[101]
	Maturity	Maturity↑	theanine, caffeine, EGCG, monoterpenes, fatty acid derivatives, total nitrogen↓, starch↑	[69,102-104,106]
Management measures	Intercropping	Cherry blossom	cis-jasmone, linalool and nonanal↑	[110]

	Walnut	Flavonoids, polyphenols, alkaloids↑, amino acid levels reduced↓	[112]
Fertilization	N fertilizer	Amino acids↑	[113]
	P–K fertilizer	Catechins↑	[113]
Pruning	Carry out pruning	Catechins, flavonoids↑	[117]

Table S2. Research details of cited literature

Article title	Research design details	Key findings
Amino acids and flavonoids analysis reveals quality constituents difference among different albino tea resources	Field sampling: 39 albinism accessions, spring-tea one-bud-two-leaves fixed in liquid nitrogen; HPLC quantification	Total free amino acids were significantly elevated while flavonoids were markedly reduced in albino tea accessions; the theanine contents of 'Huangjinyu', 'Anji-baicha', 'Jibai' and 'Fuding-dabaicha' were 1545.18, 1070.33, 899.44 and 747.57 μg/g, respectively.
A comparative metabolomic analysis reveals difference manufacture suitability in "Yinghong 9" and "Huangyu" teas	Control experiment: two cultivars of fresh leaves processed into black tea in the same factory under identical procedures; widely targeted metabolomics and GC–MS	Kaempferol-3-glucoside in 'Yinghong 9' was 1.5-fold higher than in 'Huangyu', and the kaempferol-3-glucoside / kaempferol-3-galactoside ratio was lower, indicating that 'Huangyu' is suitable for non-fermented teas such as green and white tea, whereas 'Yinghong 9' is suitable for black tea. Carotenoid profiling revealed significantly higher lutein and α-carotene in 'Yinghong 9', whereas zeaxanthin dominated in 'Huangyu' leaves.
Metabolomic and transcriptomic analyses reveal the characteristics of tea flavonoids and caffeine accumulation between Chinese and Assam varieties	Control experiment: nine tea cultivars from the same garden; widely targeted metabolomics and transcriptome sequencing	Compared with the purple-leaf 'BTZY' and green-leaf 'BTSC' groups, most flavonoids were down-regulated in the green-leaf 'MYC' cultivar, yet anthocyanin content was not the lowest; lack of TCS1 expression is the key factor causing the significant decrease in caffeine accumulation in 'MYC'.
Exploring the effect of different tea varieties on the quality of Lu'an Guapian tea based on metabolomics and molecular sensory science	Field experiment: three cultivars processed in the same factory according to Lu-an Guapian technology; molecular sensory science and metabolomics	The tea-polyphenol / amino-acid ratio of 'Shuzhaozao' (24.5) was higher than that of 'Anhui 1' (9.90) and 'Qunzhongzhong' (8.68).
Effects of temperature and light on quality-related metabolites in tea leaves	Forty-eight 3-year-old potted tea seedlings, indoor simulation of different altitudes under varying light and temperature regimes; HPLC quantification	Metabolites under simulated high-altitude conditions differed significantly from those under simulated low-altitude conditions in both intact and wounded leaves; tea quality was generally higher under simulated high altitude.
Root microbiota of tea plants regulate nitrogen homeostasis and theanine synthesis to influence tea quality	Field sampling, roots and leaves of multiple cultivars collected in autumn and spring → synthetic community inoculated into potted tea; microbial community assay, cell biology and UPLC	A microbial consortium isolated from tea roots enhanced ammonium uptake and promoted theanine synthesis.

The CsHSFA-CsJAZ6 module-mediated high temperature regulates flavonoid metabolism in Camellia sinensis	Potted tea and Arabidopsis in plant growth chambers; control experiment: HPLC and transcriptome	HPLC quantification of catechins showed that, compared with control leaves, *CsHSFA1b*-silenced plants accumulated 21 % more catechins, and *CsHSFA2*-silenced plants accumulated 17 % more, indicating that *CsHSFA1b* and *CsHSFA2* negatively regulate catechin biosynthesis.
Tea plant-legume intercropping simultaneously improves soil fertility and tea quality by changing bacillus species composition	Field paired control: tea–legume intercropping vs. monoculture, qPCR	Inter-cropped tea showed 11.6 % and 23.8 % significant reductions in tea-polyphenol and caffeine contents, respectively ($p < 0.05$), and a 20.7 % increase in leaf amino-acid content ($p < 0.05$) relative to monoculture.
Determination of catechin content in representative Chinese tea germplasms	Field sampling: 403 core accessions, spring-tea one-bud-two-leaves; HPLC quantification	Catechin contents among tea accessions ranged from 56.6 to 231.9 mg g^{-1}, averaging 154.5 ± 18.1 mg/g; the main catechin components differed significantly among provinces ($p < 0.05$).
mRNA-miRNA analyses reveal the involvement of CsbHLH1 and miR1446a in the regulation of caffeine biosynthesis in Camellia sinensis	Field sampling: five cultivars, one-bud-one-leaf stored in liquid nitrogen; LC–MS, transcriptome and small RNA analyses	miR1446a precisely targets and cleaves CsbHLH1, suppressing its expression and thereby influencing caffeine biosynthesis in tea.
Development of CAPS markers based on three key genes of the phenylpropanoid pathway in tea	Field sampling: 50 accessions; PCR-RFLP	Based on Nei's gene-diversity index, *var. sinensis* showed greater variability than *var. assamica*, and the proportion of overall diversity within cultivars was higher than that among cultivars.
Transgenic tobacco overexpressing tea cDNA encoding Dihydroflavonol 4-Reductase and Anthocyanidin Reductase induces early flowering and biotic stress tolerance	Control experiment: tobacco heterologous expression of tea DFR/ANR, wild-type vs. transgenic; phenotype and metabolite assays	Over-expression of CsDFR and CsANR cDNAs in tobacco increased flavonoid contents; total flavonoids in control tobacco were 39.8 mg quercetin-equivalent (QE) g^{-1} DW, whereas transgenic lines ranged from 47.63 to 69.77 mg QE g^{-1} DW.
Functional diversity of subgroup 5 R2R3-MYBs promoting proanthocyanidins biosynthesis and their key residues and motifs in tea plant	*Control experiment: genetic transformation*	*NtANR2* was up-regulated ~67.6-, 84.8- and 76.8-fold in *CsMYB5a/5b/5e* transgenic tobacco, respectively. NtLAR was up-regulated ~16.3-, 12.8-, 10-, 31.2- and 6-fold in *CsMYB5a/5b/5e/5f/5g* transgenic tobacco. Moreover, CsMYB5a/b/e directly activated the promoters of CsLAR and CsANR, whereas CsMYB5f/g activated only the CsLAR promoter.
Genome assembly of wild tea tree DASZ reveals pedigree and selection history of tea varieties	Field sampling: SMRT on wild ancient trees; RNA-seq on 217 tea accessions; transient over-expression of CsANR, CsF3′5′H and CsMYB5 and enzymatic assays	Favourable alleles or haplotypes for catechin content were identified.

Comparative transcriptomic analysis reveals the regulatory mechanisms of catechins synthesis in different cultivars of Camellia sinensis	Field sampling: one-bud-one-leaf and second leaf of two cultivars; HPLC and DNBSEQ	Two hundred and twelve single genes encoding 13 key enzymes involved in catechin biosynthesis were identified; spatial models of leucoanthocyanidin reductase and anthocyanidin reductase structures were constructed.
Expression Analysis and Functional Identification of CsTT2 R2R3-MYB Transcription Factor in Tea Plants	Control experiment: field sampling of one-bud-two-leaves from three tea cultivars; phylogenetic analysis, gene-expression pattern analysis and molecular biological tests	CsTT2 localises to the nucleus, encodes a transcriptional activator, and binds the ANR promoter to activate catechin-biosynthetic genes.
Phenylalanine ammonia-lyase (PAL) and cinnamate 4-hydroxylase (C4H) and catechins (flavan-3-ols) accumulation in tea	Field sampling: one clonal cultivar, apical bud, first to fourth leaves; RT-PCR	Catechin contents decreased by 22 %, 21 % and 13 % under drought stress (DS), abscisic acid (ABA) and gibberellic acid (GA_3) treatments, respectively, compared with day 0, but increased by 15 % and 20 % at 12 h and 24 h after wounding, respectively.
Identification of a Flavonoid Glucosyltransferase Involved in 7-OH Site Glycosylation in Tea plants	In-vitro control: spring-tea seedlings, buds and tender roots plus Arabidopsis; site-directed mutagenesis and enzymatic assays	Single- and triple-substitution assays confirmed that residues P238, T239 and F240 may functionally substitute or compensate for flavonoid 7-O-glucosyltransferase activity.
Insights into acylation mechanisms: co-expression of serine carboxypeptidase-like acyltransferases and their non-catalytic companion paralogs	Control experiment: leaves collected in garden from C. sinensis L. cv. 'Shuchazao', C. oleifera Abel., V. vinifera L. and persimmon; transgenic plants, recombinant enzymes and gene mutation	CsSCPL4 is a catalytic acyltransferase, whereas CsSCPL5 is a non-catalytic companion paralogue (NCCP); co-expression of CsSCPL4 and CsSCPL5 likely accounts for galloylation.
Tissue-specific, development-dependent phenolic compounds accumulation profile and gene expression pattern in tea plant	Field sampling: young shoots (bud, first–fourth leaves, tender stem) and tender roots of tea; LC-MS/MS qualification, real-time PCR and cluster analysis	Phenolic accumulation in tea is developmentally regulated in buds, leaves and roots, with higher contents in young leaves; expression patterns of genes in the C2-2-1 and C2-2-2-1 groups may underlie this developmental dependence.
CsMYB1 integrates the regulation of trichome development and catechins biosynthesis in tea plant domestication	Field sampling: trichome-enriched tea cultivar for gene cloning, phenotype survey, transcriptome and metabolomics; potted Arabidopsis for transformation assays	The CsMYB1-MBW complex regulates trichome-development genes *CsGL2* and *CsCPC*, and key galloylated catechin-biosynthetic genes *CsANR* and *CsSCPL1A*.

Discovery and characterization of tannase genes in plants: roles in hydrolysis of tannins	Field sampling: leaves of Camellia sinensis (Cs), Vitis vinifera (Vv), Juglans regia (Jr), Citrus clementina (Cc), Diospyros kaki (Dk) and Fragaria × ananassa (Fa) for gene cloning and expression; transcriptional and metabolic analyses	CsTA expression is inversely correlated with galloylated catechin accumulation.
Association analysis of BSA-seq, BSR-seq, and RNA-seq reveals key genes involved in purple leaf formation in a tea population	Control experiment: field sampling of 30 extreme-colour (dark-purple vs. green) individuals; RNA-seq, BSA-seq and BSR-seq analyses	Two highly expressed genes (CsANS and CsMYB75) carrying SNP/InDel variants in purple-leaf tea positively regulate anthocyanin accumulation; a 181-bp InDel in the CsMYB75 promoter co-segregates with leaf colour.
A light responsive transcription factor CsbHLH89 positively regulates anthocyanidin synthesis in tea	Control experiment: two purple-tea cultivars sampled in field; HPLC, qRT-PCR, yeast one-hybrid, yeast two-hybrid	CsbHLH89 binds the G-box element and activates CsCHS, CsFLS and CsDFR promoters *in vitro* and *in vivo*, positively regulating anthocyanin synthesis; CsHY5 activates CsbHLH89 expression.
Insight into the pigmented anthocyanins and the major potential co-pigmented flavonoids in purple-coloured leaf teas	Field sampling: nine purple-leaf tea cultivars harvested one-bud-two-leaves, widely targeted metabolomics	Four purple-leaf teas contained high levels (>10 mg g^{-1}) of (−)-epigallocatechin-3-(3″-O-methyl)gallate.
Analysis of Differentiated Chemical Components between Zijuan Purple Tea and Yunkang Green Tea by UHPLC–Orbitrap–MS/MS	Field sampling: two cultivars processed into green tea, UHPLC-Orbitrap-MS/MS, HPLC	'Zijuan' likely produces anthocyanins and proanthocyanidins via flavonol and flavone glycosides; concurrent increases in both contribute to its unique purple colour.
Metabolic analyses reveal different mechanisms of leaf color change in two purple-leaf tea plant cultivars	Field sampling: two purple tea cultivars; metabolome and transcriptome	Leaf-colour change in 'Zixuan' is mainly due to decreased flavonoid/anthocyanin contents, coupled with increased porphyrin and chlorophyll metabolism, carotenoid biosynthesis and steroids, and decreased fatty acids.
CsCHLI plays an important role in chlorophyll biosynthesis of tea plant	Control experiment: one albino and two green tea cultivars sampled in greenhouse; transcriptome, transgenic Arabidopsis, yeast two-hybrid	Over-expression of CsCHLI restored the chlorophyll-deficient phenotype of the *atchli1 Arabidopsis* mutant; chloroplast-localised CsCHLI interacts with CsCHLD and CsCHLH to form a heterocomplex essential for chlorophyll biosynthesis.
Conversion obstacle from Mg-protoporphyrin IX to protochlorophyllide might be responsible for chlorophyll-deficient phenotype of Huangjinya's albino offspring	Field sampling: albino cultivar HJY (female parent), 16 four-year-old progenies (HJY1–HJY16) and normal green male parent 'Fuding-dabaicha' (FD); HPLC, cell biology	Low chlorophyll in albino progeny shoots results mainly from impaired conversion of Mg-protoporphyrin IX to Pchlide, likely due to down-regulation of CRD and DVR.

Characterization of l-Theanine Hydrolase in Vitro and Subcellular Distribution of Its Specific Product Ethylamine in Tea	Control experiment: different leaf-colour tea cultivars sampled in field; metabolome and transcriptome	CsPDX2.1, cloned with its *Arabidopsis* homologue, catalyses hydrolysis of l-theanine to ethylamine and l-glutamate *in vitro*; CsPDX2.1 transcripts are higher in green tissues and lower in albino (yellow) tea.
Transcriptomic and biochemical analysis reveal differential regulatory mechanisms of photosynthetic pigment and secondary metabolites between high amino acids green-leaf and albino tea cultivars	*Control experiment: four tea cultivars sampled in field, HPLC and transcriptome*	*CsGOGAT* and *CsGS/TS* are the key genes responsible for the differential l-theanine accumulation patterns between 'Huangjinya 2' and 'Baojing-Huangjinya 1'.
Differential Accumulation of Aroma Compounds in Normal Green and Albino-Induced Yellow Tea Leaves	Field sampling: 'Yinghong 9' and its yellow mutant; GC-MS and transcriptome	Lower linalool in albino-induced yellow leaves compared with normal green leaves is due to reduced GDP content.
Differential metabolites and their transcriptional regulation in seven major tea cultivars	Field sampling: seven tea cultivars, widely targeted metabolome and transcriptome	Purple and green shoots of 'Tie-guanyin' arise from anthocyanin accumulation and down-regulation of Mg-protoporphyrin IX monomethyl ester cyclase (MPEC) (CsTGY10G0001989).
Transcriptome and Metabolite Profiling Reveal Novel Insights into Volatile Heterosis in the Tea Plant	Control experiment: four tea cultivars (parents and hybrids) sampled in field; RNA-seq and TD-GC-MS	Thirty-three TFs highly correlated with volatile genes showed differential expression in the hybrid compared with either parent.
Natural allelic variations of TCS1 play a crucial role in caffeine biosynthesis of tea plant and its related species	Field sampling: 60 representative accessions plus control 'Fuding-dabaicha'; transcriptome and HPLC	Two molecular mechanisms underlie low-caffeine accumulation: low-transcription-level TCS1 alleles or a TCS1-encoded protein with only TS activity.
CsMYB184 regulates caffeine biosynthesis in tea plants	Control experiment: eleven tea cultivars; transcriptome and cell biology	An LTR insertion in the MYB184 promoter of wild tea *C. ptilophylla* explains its low TCS1 expression and caffeine content.
The transcription factor CsS40 negatively regulates TCS1 expression and caffeine biosynthesis in connection to leaf senescence in Camellia sinensis	Control experiment: yeast one-hybrid, split-luciferase, EMSA, sub-cellular localization, qRT-PCR, gene knock-down/over-expression and metabolite profiling	The senescence-associated factor CsS40 negatively regulates TCS1 and suppresses caffeine accumulation in 'Fuding-dabaicha'.
L-Theanine Content and Related Gene Expression: Novel Insights into Theanine Biosynthesis and Hydrolysis among Different Tea Plant Tissues and Cultivars	Field sampling: buds and first, second, third, old leaves, new stems and lateral roots of three cultivars; RP-HPLC and transcriptome	Seventeen genes encoding theanine-metabolic enzymes were identified: CsTS1, CsTS2, CsGS1, CsGS2, CsGOGAT-Fe, CsGOGAT-NAD(P)H, CsGDH1, CsGDH2, CsALT, CsSAMDC, CsADC, CsCuAO, CsPAO, CsNiR, CsNR, CsGGT1 and CsGGT3. Transcriptional profiles in different tissues of three cultivars revealed significantly higher transcript levels of most selected genes in 'Huangjinyu' than in 'Anji-baicha' and 'Yingshuang'. CsTS2, CsGS1 and

		CsGDH2 transcript levels were positively correlated with theanine content, whereas the others were negatively correlated.
Acetylation participation in theanine biosynthesis: Insights from transcriptomics, proteomics, and acetylomics	Control experiment: three tea cultivars sampled in field; transcriptomics, proteomics and acetylomics	Theanine content correlated positively with GS, GOGAT, GDH and AlaDC, and negatively with TS.
CsAlaDC and CsTSI work coordinately to determine theanine biosynthesis in tea plants	Tobacco transient expression	Theanine biosynthesis requires ethylamine and the synergistic action of CsAlaDC and CsTSI.
Transcriptomic analyses reveal variegation-induced metabolic changes leading to high L-theanine levels in albino sectors of variegated tea	Field sampling: green, albino and mosaic sectors of 'Yanling-Huayecha'; RNA-seq, HPLC and cell biology	Down-regulation of CsPPOX1, CsLHCB6, CsFdC2 and CsSCY1, together with thylakoid damage, causes variegation; up-regulation of CsTSI and CsAlaDC may enhance theanine synthesis.
CsAAP7.2 is involved in the uptake of amino acids from soil and the long-distance transport of theanine in tea plants	Control experiment: first, third, fifth leaves, flowers, stems and roots of tea cuttings in climate chamber; transcriptome and cell biology	Two new CsAAP homologues, CsAAP7.1 and CsAAP7.2, were identified; CsAAP7.2 transports theanine and other amino acids, whereas CsAAP7.1 lacks transport activity.
CsMYB73 negatively regulates theanine accumulation mediated by CsGGT2 and CsGGT4 in tea shoots	Control experiment: two-year-old hydroponic cuttings of 'Shuchazao' and 'Huangkui' in greenhouse; cell biology and enzyme assays	γ-Glutamyltranspeptidase CsGGT4, a homologue of the theanine-hydrolase CsGGT2, exhibits higher catalytic efficiency for theanine synthesis; sustained increase of CsMYB73 significantly induces CsGGT2 and suppresses CsGGT4 expression.
Metabolomics and Transcriptomics Analyses Reveal Nitrogen Influences on the Accumulation of Flavonoids and Amino Acids in Young Shoots of Tea Plant	Field trial: nitrogen deprivation, nitrate, ammonium and nitric oxide; metabolomics and transcriptomics	Nitrogen deficiency accumulated various flavonoids, corresponding to higher expression of hub genes F3H, FNS, UFGT, bHLH35 and bHLH36.
Nitrogen-Regulated Theanine and Flavonoid Biosynthesis in Tea Plant Roots: Protein-Level Regulation Revealed by Multiomics Analyses	Hydroponic control: nitrogen deficiency vs. normal nitrogen; proteome, ubiquitome, transcriptome	Nitrogen deficiency inhibited root theanine metabolism and promoted flavonoid metabolism; theanine content was 30.316 ± 1.805 mg g^{-1} under N deficiency vs. 56.840 ± 2.846 mg g^{-1} in the control.

Effects of Long-Term Nitrogen Fertilization on the Formation of Metabolites Related to Tea Quality in Subtropical China	Control experiment: four N applications (N0, N1, N2, N3) in garden; UPLC–QTOF-MS and transcriptome	Long-term nitrogen fertilisation up-regulated chlorophyll-biosynthetic genes, increased chlorophyll and yield, and markedly raised free amino acids—especially l-theanine—while reducing catechin contents, improving liquor freshness. However, it significantly decreased benzyl alcohol and 2-phenylethanol in fresh leaves, and (E)-nerolidol and indole in withered leaves, impairing floral and fruity aroma formation.
CsPHRs-CsJAZ3 incorporates phosphate signaling and jasmonate pathway to regulate catechin biosynthesis in Camellia sinensis	Hydroponic control: low vs. sufficient phosphorus on hydroponic tea; RNA-seq, HPLC and cell biology	CsPHR1 and CsPHR2, two phosphate-signalling TFs, regulate catechin biosynthesis by activating *CsANR1* and *CsMYB5c* transcription; the JA repressor CsJAZ3 negatively regulates catechin biosynthesis via physical interaction with CsPHR1 and CsPHR2.
K-solubilizing bacteria (Bacillus) promote theanine synthesis in tea roots by activating CsTSI activity	Control trial: pot and in-vitro enzyme assays; metabolome, transcriptome and proteome	Increasing potassium levels activated recombinant CsTSI activity and elevated ethylamine (theanine precursor), thereby promoting root theanine synthesis.
Dynamic Effects of Excessive Calcium on Biochemical Components in Young Shoots and Root Growth of Tea	Hydroponic trial: Ca^{2+} gradient (30, 90, 150, 210, 270 mg L^{-1}); HPLC	Under excess calcium, tea-polyphenol contents increased during the first two weeks at 90 and 150 mg L^{-1}, likely as an antioxidant response, whereas amino-acid contents declined from the third week onward; caffeine decreased from the first week and continued to decline with treatment duration.
Effect of root zone pH and form and concentration of nitrogen on accumulation of quality-related components in green tea	Hydroponic precision control: pH 4.0–6.0 × ammonium/nitrate ratio; metabolome	Caffeine concentrations increased in NH_4^+- and ($NH_4^+ + NO_3^-$)-supplied plants, whereas catechins decreased in ($NH_4^+ + NO_3^-$)-fed plants. Root-zone pH did not influence most free amino acids except theanine, which increased at low pH (4.0) regardless of N form, probably due to an accumulation effect as growth declined more strongly than N absorption. Raising N supply increased N allocation to free amino acids, most strikingly to arginine.
Effects of Bio-organic Fertilizer on Soil Fertility, Yield, and Quality of Tea	Field trial: five treatments: no fertilizer, Bacillus megaterium bio-organic, B. mucilaginosus bio-organic, B. subtilis bio-organic and conventional chemical fertilizer; metabolome and enzyme assays	Compared with conventional chemical fertiliser, bio-organic fertilisers based on *Bacillus megaterium*, *B. mucilaginosus* and *B. subtilis* increased tea-polyphenol, amino-acid and caffeine contents by 17.71 %, 33.05 % and 22.20 %, respectively, and significantly enhanced soil leucine aminopeptidase, β-glucosidase, β-N-acetylglucosaminidase, acid phosphatase, β-cellobiosidase and β-xylanase activities ($p < 0.05$).

CsMYBL2 homologs modulate the light and temperature stress-regulated anthocyanin and catechins biosynthesis in tea plants	Pot control: hydroponic seedlings, light intensity × temperature; metabolome, transcriptome and cell biology	The CsbZIP1-CsmiR858a-CsMYBL2 module mediates UV-B- or cold-activated regulation of anthocyanin/catechin biosynthesis by CsMYB75/CsMYB86 via repression of CsMYBL2a and CsMYBL2b. Likewise, the CsCOP1-CsbZIP1-CsPIF3 module and BR signalling mediate high-temperature suppression of anthocyanin and catechin biosynthesis by differentially up-regulating CsMYBL2b and CsMYBL2a, respectively.
CsGOGAT Is Important in Dynamic Changes of Theanine Content in Postharvest Tea Plant Leaves under Different Temperature and Shading Spreadings	*Control experiment: temperature (4 °C, 25 °C, 38 °C) × shading (0 %, 100 %); enzyme assays + HPLC*	CsFd-GOGAT transcript levels correlated positively with theanine content, whereas CsNADH-GOGAT correlated negatively. Except under shading, CsFd-GOGAT protein levels correlated negatively and CsNADH-GOGAT positively with theanine content.
Increasing Temperature Changes Flux into Multiple Biosynthetic Pathways for 2-Phenylethanol in Model Systems of Tea and Other Plants	Control trial: C. sinensis cv. 'Jinxuan', Solanum lycopersicum cv. 'Micro-Tom' and Petunia × hybrida cv. 'Mitchell Diploid'; metabolome, transcriptomics, chemical synthesis, isotope-tracer feeding and cell biology	Elevated temperature increased L-Phe flux into the 2-phenylethanol pathway via PPA and PAld.
Characterization of the Difference between Day and Night Temperatures on the Growth, Photosynthesis, and Metabolite Accumulation of Tea Seedlings	Phytotron: one-year-old seedlings, diurnal temperature differences 5/10/15 °C, 15/30/45 d; genome, transcriptome and metabolome	DIF 10 °C yielded higher caffeine and amino-acid contents but lower soluble sugars, tea-polyphenols and catechins, probably due to high expression of amino-acid and N-metabolism genes and low expression of flavonoid-biosynthetic genes.
昼夜温差对茶鲜叶挥发性及非挥发性品质成分及相关生理指标的影响	Control experiment: four diurnal temperature differences, two-year-old seedlings in phytotron; GC-MS + HPLC	Free amino-acid and caffeine contents increased significantly with larger DIF, reaching 2.44- and 2.55-fold at 15 °C DIF compared with 0 °C DIF. Total flavonoids and the phenol/amino-acid ratio decreased significantly ($P < 0.05$), reaching minima of 2.84 % and 3.24 at 15 °C DIF. Free amino-acid contents correlated extremely significantly positively with Pn, whereas the phenol/amino-acid ratio correlated negatively ($P < 0.01$).
昼夜温差对茶树生长及茶叶品质的影响	Control experiment: three diurnal temperature differences, one-year-old seedlings in phytotron; metabolome	DIF 10 °C for 30 d gave 2.2 % theanine in young shoots, 15 % higher than DIF 0 °C, with no significant difference in EGCG.
CsbZIP1-CsMYB12 mediates the production of bitter-tasting flavonols in tea plants through a coordinated activator–repressor network	Control experiment: five tea cultivars under shading; UPLC, transcriptome, cell biology	UV-B activation and shading suppression of flavonol biosynthesis are coordinated by a complex network in which CsbZIP1 and CsPIF3 act as positive MYB activator and negative MYB repressor, respectively.

Responses of secondary metabolites and transcriptomes in the tea cultivar ‘Zhong Ming 6’ to blue light and red light	Control trial: plant growth chamber, light quality (red, blue, white); HPLC and transcriptome	CsMYB4 expression under red light was highly significantly and positively correlated with total catechin and anthocyanin accumulation.
Red Light Regulates the Metabolite Biosynthesis in the Leaves of “Huangjinya” Through Amino Acid and Phenylpropanoid Metabolisms	Light-quality control: two-year-old ‘Huangjingya’ in greenhouse, light quality (red, white); transcriptome + LC-MS	Consistent with transcriptomics, red-light supplementation enriched most differential metabolites in amino acids, increasing sweet and umami amino acids while decreasing bitter amino acids.
Ambient Ultraviolet B Signal Modulates Tea Flavor Characteristics via Shifting a Metabolic Flux in Flavonoid Biosynthesis	Control trial: two garden cultivars, light (natural vs. shaded); metabolome, transcriptome, cell biology	UV-B deficiency decreased bitter and astringent flavonol glycosides (kaempferol-7-O-glucoside, myricetin-3-O-glucoside and quercetin-7-O-glucoside) but increased non-galloylated catechins. Conversely, UV-B supplementation increased flavonols and decreased catechins. These responses are mediated by CsHY5, which induces MYB12 activation and binds promoters of flavonoid-biosynthetic genes (CsFLS, CsLARa and CsDFRa).
Regulation of formation of volatile compounds of tea leaves by single light wavelength	Control trial: light (blue, red, natural); metabolome, transcriptome, cell biology, enzyme assays	Blue and red lights significantly up-regulated 9/13-lipoxygenases involved in VFADs, phenylalanine ammonia-lyase involved in VPBs, and terpene synthases involved in VTs. Single wavelengths had less influence on postharvest leaf volatiles than on preharvest leaves.
Transcriptomic Analysis Reveals the Molecular Mechanisms of Drought-Stress-Induced Decreases in Camellia sinensis Leaf Quality	Control trial: two-year-old tea in phytotron; metabolome, transcriptome	Drought stress significantly increased total flavonoids but decreased caffeine and theanine accumulation.
Transcriptome and metabolome profiling unveiled mechanisms of tea quality improvement by moderate drought on pre-harvest shoots	Control trial: three-year-old ‘Yunkang 10’ under rain-out shelter: −20 % precipitation; metabolome and transcriptome	Drought markedly increased isoflavonoids and glycosylated flavonoids and sharply decreased lipids, mainly due to strong up-regulation of type-III polyketide synthase B (PKSB), flavonol synthase/flavanone 3-hydroxylase (FLS) and UDP-glycosyltransferases (UGTs), significant suppression of anthocyanidin synthase (ANS) and R2R3MYB, and down-regulation of lipid metabolism.

Impact of Mild Field Drought on the Aroma Profile and Metabolic Pathways of Fresh Tea Leaves Using HS-GC-IMS and HS-SPME-GC-MS	Control trial: eight-year-old 'Fuding-dabaicha' (FD) and 'Wuniuzao' (WNZ) subjected to drought in garden	Drought-stressed plants showed significantly higher VOC concentrations and diversity, with aroma notes related to sweet, fruity, caramel, floral and earthy scents, whereas green, grassy, leafy, woody, herbal and floral notes decreased. Notably, methylated compounds, aromatic oxides and medium-/long-chain VFADs increased, whereas sesquiterpenes decreased markedly. Cultivars responded differently: FD-D exhibited increased VOC diversity, whereas WNZ showed greater increases in low-threshold VOC concentrations.
Effects of geographic locations and topographical factors on secondary metabolites distribution in green tea at a regional scale	Regional-scale field: 78 local accessions sampled in southern Jiangsu; metabolome	Northern regions had higher TC, EGCG+GCG, ECG+CG contents and ECG/EC ratios but lower free amino acids, resulting in stronger bitterness and astringency. Hill-grown teas, receiving shorter and weaker sunlight than plain-grown teas, accumulated more free amino acids due to a natural shading effect, whereas plains teas accumulated more TC.
Comprehensive analysis of environmental factors on the quality of tea fresh leaves	Multi-site field: soil and leaf samples from 27 gardens in Wanzhai township, Xuan'en county, Enshi city, Hubei; metabolome and soil physico-chemical analyses	With increasing altitude, amino acids increased gradually, catechins first decreased then increased, phenolic acids varied with catechins, and flavonol (alcohol) glycosides showed little change.
Striking changes in tea metabolites due to elevational effects	Field sampling: two mountain ranges at different altitudes in Yunnan; metabolome	Multivariate ANOVA showed that low-altitude teas had statistically ($p = 0.0062$) higher concentrations of bitter compounds—caffeine, epicatechin gallate, gallocatechin and catechin.
Integrated transcriptomics and metabolomics analysis of catechins, caffeine and theanine biosynthesis in tea plant over the course of seasons	Field sampling; seasons (spring, summer, autumn); metabolome and transcriptome	Theanine abundance was significantly higher in spring than in summer and autumn, whereas caffeine showed no seasonal difference.
Transcriptomic Insights into the Enhanced Aroma of Guangdong Oolong Dry Tea in Winter	Field sampling: seasons (spring, winter); metabolome and transcriptome	UDP-glycosyltransferase genes were significantly up-regulated in winter fresh leaves, probably promoting terpene-glycoside synthesis.
Characterization of CsTSI in the Biosynthesis of Theanine in Tea Plants	Field sampling: buds, first leaves, stems, roots, flowers and fruits; metabolome, transcriptome, cell biology	*RNAi* knock-down or over-expression of *CsTSI* in tea hairy roots decreased or increased theanine and glutamine contents, respectively. Recombinant CsTSI used glutamate as acceptor and ammonium or EA as donors to synthesise glutamine and theanine.
Glutamine Synthetases Play a Vital Role in High Accumulation of Theanine in Tender Shoots of Albino Tea Germplasm	Field sampling: young leaves, old leaves, stems and roots collected from 2-year-old C. sinensis cv. Fudingdabai (FDDB) and 3-year-old C. sinensis cv.;	In white tender buds, cytosolic CsGS1.2 expression increased to compensate for reduced chloroplastic CsGS2, which plays a crucial role in high theanine accumulation in 'Huabai 1'.

	metabolome, transcriptome, cell biology, enzyme assays	
Transcriptomic analysis reveals molecular adaptation of secondary metabolic pathways to multiple macronutrient starvation in tea	Hydroponic nutrient omission: one-year-old Longjing 43; N/P/K starvation; transcriptome and metabolome	Under potassium deficiency, the most complex expression profile with the greatest number of altered genes indicated a stronger response of the catechin-biosynthetic pathway to K than to other nutrients. Under N starvation, only GDH and AIDA were down-regulated, indicating insensitivity of the theanine pathway to N deficiency. In contrast, K deficiency caused the greatest changes in TS, GS, GDH and AIDA expression, followed by P deficiency, which affected GS, GOGAT and GDH genes.
Differential regulatory mechanisms of secondary metabolites revealed at different leaf positions in two related tea cultivars	Field sampling: two cultivars, leaves of different maturity; metabolome and transcriptome	In ‘Zhongcha 108’, more active catechin biosynthesis suppressed N-rich metabolites, especially theanine, whereas in ‘Zhongming 7’ the balance shifted toward increased N-containing compounds (theanine and caffeine).
Identification of UDP-glycosyltransferases involved in astringent tastecompounds	A. membranaceus in plant growth chamber; metabolome, transcriptome, cell biology, enzyme kinetics	AmUGT88E29 and AmUGT88E30 exhibited catalytic activity toward multiple flavonoids.
Global transcriptional analysis of catechins, caffeine and theaninebiosynthesis over seasons	Field sampling (spring, summer, autumn); metabolome and transcriptome	Catechin abundance was higher in summer than in spring and autumn, whereas theanine was significantly higher in spring; caffeine showed no seasonal change. Photosynthesis-pathway genes were significantly down-regulated, probably contributing to seasonal differences in phenotype and metabolites.
Synergistic effects of pre-harvest drought and shade on flavorenhancement	Field sampling: control, drought, shading and drought + shading groups; electronic-tongue analysis, physiological-biochemical analysis and metabolome	Jasmonic-acid and abscisic-acid signalling pathways activated aroma accumulation under drought + shading treatment.
Molecular characterization of WRKY transcription factors that negativelyregulate O-methylated catechin biosynthesis	Control experiment: three different garden cultivars; HPLC, transcriptome and cell biology	CsWRKY31 and CsWRKY48 repress transcription of *CsLAR*, *CsDFR* and *CCoAOMT* by binding to W-box elements in their promoters.
